# Contribution towards a Metabolite Profile of the Detoxification of Benzoic Acid through Glycine Conjugation: An Intervention Study

**DOI:** 10.1371/journal.pone.0167309

**Published:** 2016-12-01

**Authors:** Cindy Irwin, Mari van Reenen, Shayne Mason, Lodewyk J. Mienie, Johan A. Westerhuis, Carolus J. Reinecke

**Affiliations:** 1 Centre for Human Metabolomics, Faculty of Natural Sciences, North-West University (Potchefstroom Campus), Potchefstroom, South Africa; 2 Department of Statistics, Faculty of Natural Sciences, North-West University (Potchefstroom Campus), Potchefstroom, South Africa; 3 Biosystems Data Analysis, Swammerdam Institute for Life Sciences, University of Amsterdam, Amsterdam, The Netherlands; California State University Fresno, UNITED STATES

## Abstract

Benzoic acid is widely used as a preservative in food products and is detoxified in humans through glycine conjugation. Different viewpoints prevail on the physiological significance of the glycine conjugation reaction and concerns have been raised on potential public health consequences following uncontrolled benzoic acid ingestion. We performed a metabolomics study which used commercial benzoic acid containing flavored water as vehicle for designed interventions, and report here on the controlled consumption of the benzoic acid by 21 cases across 6 time points for a total of 126 time points. Metabolomics data from urinary samples analyzed by nuclear magnetic resonance spectroscopy were generated in a time-dependent cross-over study. We used ANOVA-simultaneous component analysis (ASCA), repeated measures analysis of variance (RM-ANOVA) and unfolded principal component analysis (unfolded PCA) to supplement conventional statistical methods to uncover fully the metabolic perturbations due to the xenobiotic intervention, encapsulated in the metabolomics tensor (three-dimensional matrices having cases, spectral areas and time as axes). Identification of the biologically important metabolites by the novel combination of statistical methods proved the power of this approach for metabolomics studies having complex data structures in general. The study disclosed a high degree of inter-individual variation in detoxification of the xenobiotic and revealed metabolic information, indicating that detoxification of benzoic acid through glycine conjugation to hippuric acid does not indicate glycine depletion, but is supplemented by ample glycine regeneration. The observations lend support to the view of maintenance of glycine homeostasis during detoxification. The study indicates also that time-dependent metabolomics investigations, using designed interventions, provide a way of interpreting the variation induced by the different factors of a designed experiment–an approach with potential to advance significantly our understanding of normal and pathophysiological perturbations of endogenous or exogenous origin.

## Introduction

Applications of metabolomics to intervention or challenge studies greatly enhance the holistic understanding of the effects of consumed substances on metabolic pathways [1, 2]. Data sets from intervention studies are, however, complex, as these investigations aspire to measure multiple metabolites in a biofluid, obtained from several experimental subjects, collected at different points in time and subjected to interventions from different consumed substances. In addition, these studies call for methods of data analysis specifically designed for longitudinal (time-dependent), multi-subject (data from several experimental participants), multi-group (intervention studies) and multivariate data [3, 4]. In this paper, we present the experimental design for an intervention study which includes the complex aspects mentioned above. The interventions were consumption of alcohol in the presence or absence of NAD, using flavored water as a vehicle. We generated matched-sample series through a cross-over study of participating subjects, collecting samples over a distinct time frame. The biochemical responses to the interventions were distinctly different: responses to alcohol and NAD intake resided in the intermediary metabolism, whereas those to exogenous substances in the vehicle involved detoxification through biotransformation mechanisms. Here, we present the full experimental design of the intervention study, but focus on the contribution from the biotransformation response by presenting the outcomes of vehicle consumption only. The results on the alcohol and NAD interventions will be published in a separate paper.

Benzoic acid was an important constituent in the vehicle used in the present study. Benzoic acid and its derivatives are routinely used as preservatives and flavoring agents in food products. Consequently, human exposure to them is quite common, and has raised concerns about potential public health consequences [5]. Evidence that benzoic acid is excreted as hippuric acid after enzymatic conjugation to glycine dates back to the 1950s [6], but different viewpoints seem to prevail on the physiological significance of the glycine conjugation reaction. Traditionally, glycine conjugation became part of the paradigm of detoxification, with the critical role of glycine conjugation for aromatic acids [7]. More recently, new views were proposed, shifting the focus to glycine homeostasis to assist in the regulation of body stores of glycine and other amino acids which are key neurotransmitters in the central nervous system (CNS) [8] or to serve as a molecular escort in the glycine deportation system to excrete excess glycine into urine as hippuric acid [9].

Detoxification pathways–the traditional viewpoint–can directly affect the integrity of multiple organs and hence can be widely involved in a variety of human conditions, such as health [10], co-metabolism in humans with the gut microbiome [11], disease therapy [12] and aging [13]. Lipophilic endogenous or exogenous xenobiotics are first metabolized by the phase 1 detoxification system, which converts the compounds into substances having a hydrophilic functional group for increased solubility. The phase 2 detoxification system involves conjugation reactions [14]. The phase 2 system comprises several enzymes, with glycine-N-acyltransferase (GLYAT) being the key enzyme in glycine conjugation.

Invoking the roles of aromatic acids and gut metabolites to regulate blood levels of glycine–the new viewpoint–relates to that of glycine as neurotransmitter in the CNS. A putative role for it as a neurotransmitter dates back to the observation that the concentration of glycine in spinal cord tissue is far higher than elsewhere in the brain [15]. The intracellular concentration of glycine is regulated mainly by a glycine transporter, acting in synergy with glutamic acid decarboxylase [16]. The proposed glycine deportation system, moreover, functions as a homeostatic regulator of the GLY central pool and, by definition, also the GLY tissue pool [9].

GLYAT (reviewed by Badenhorst *et al*. [17]) is located in the mitochondria of mammalian liver and kidney and is a member of the superfamily of N-acyl-transferases. It has been demonstrated that several GLYAT species [glycine N-acyltransferase (EC 2.3.1.13), glutamine N-acyltransferase (EC 2.3.1.68) and glycine N-benzoyltransferase (EC 2.3.1.71)] are closely related but distinct mitochondrial enzymes [18], although originally regarded as being a single enzyme [19]. Benzoyl-CoA, salicyl-CoA, and certain short-, straight- and branched-chain fatty acyl-CoA esters are substrates for the former enzyme, and glycine is the acyl acceptor for all these enzymes. As glycine and glutamic acid are known to cross the blood–brain barrier freely, it is assumed that removal of GLY from the GLY central pool will lower levels of GLY in the CNS [9].

Commercial flavored water, containing benzoic acid (the preservative and co-substrate in glycine conjugation), was used as vehicle for the present intervention study, which opened the opportunity for a metabolomics investigation on controlled benzoic acid consumption. The intervention study consisted of 24 experimental cases (healthy males between 20 and 24 years of age), and used a metabolomics approach for the experimental design and data analysis. The study included four treatments and six urine samples per experimental case were collected over a period of five hours and used as biofluid for the metabolomics measurements. We selected proton nuclear magnetic resonance (^1^H-NMR) spectroscopy as the technology for the generation of the metabolomics data as it is the recognized method of choice for an untargeted coverage of the metabolome. The outcomes offered several intriguing views on the metabolites formed in each of these interventions. The results using the vehicle as control provided novel insights into the metabolite profile following benzoic acid consumption.

Time-dependent intervention studies require more technical statistical methods for data analysis, forming another central aspect of this paper. Metabolomics data are mostly represented as a matrix of controls and cases (rows) measured over the metabolites that reflect the perturbation under investigation (columns). Such data are traditionally processed through multivariate statistical methods, using mostly unsupervised principal component analysis (PCA) and a supervised method such as partial least-squares discriminant analysis (PLS-DA), to identify metabolites that differ significantly between the test groups studied [20]. In human intervention studies, individual variation tends to dominate the often less pronounced metabolic perturbations due to the intervention. A reasonably large number of cases is mostly required as well as a sufficient number of time points to cover the effect of the intervention. We used a cross-over experimental design, which generated several hundred samples which requires a different approach from the traditional multivariate methods for processing the data and a more extensive validation of the results than for standard metabolomics studies.

For optimal insight into the intervention involving vehicle consumption, we followed a novel approach of complementary statistical methods: (1) the traditional multivariate and univariate methods of analysis were included as the first approximation of the biological profile following the intervention; (2) ASCA (ANOVA-simultaneous component analysis), developed for analyzing designed metabolomics data [4], was used next as a method that deals with multivariate data sets based on a defined experimental design, including time-dependent data; (3) RM ANOVA (repeated measures analysis of variance), which is similar to ASCA as it accounts for the experimental design of the data (although RM ANOVA was used to assess each metabolite individually to find those metabolites that changed significantly with time); finally, (4) unfolded PCA [21] was applied to gain insight into the global (i.e. multivariate) effect of the vehicle over time. The combination of these complementary statistical methods and transdisciplinary approach of metabolomics to the research lead to a more holistic view of the data. This proved essential in elucidating the effect of the intervention and provided novel insights into benzoic acid biotransformation, not observed by more reductionist methods of analysis.

## Materials and Methods

### Chemicals and reagents

The substances used for the intervention reported here were flavored water (aQuellé lemon-flavored sparkling water: fructose and citric acid flavoring; sodium benzoate preservative; sodium cyclamate, aspartame, acesulfame K sweeteners; vitamin C– www.aquelle.co.za, product of South Africa), commercial water (Valpré still spring water, inorganic contents specified–product of South Africa). The internal standard for the ^1^H-NMR analysis was trimethyl-2,2,3,3-tetradeuteropropionic acid (TSP, sodium salt; Sigma Aldrich).

### Experimental design

The intervention study was based on an observation in a preliminary metabolomics investigation on the effect of acute alcohol consumption in healthy cases [22] that indicated some NAD depletion. The experimental group consisted of 24 medically confirmed healthy males, between 20 and 24 years of age, residing in the same student hostel of North-West University (South Africa). They were neither alcohol addicts nor total abstainers, but confirmed their use of alcohol on a moderate social level. No cases took any medication, all were asked to refrain from vitamins, minerals, and other supplementation, and were requested to follow a similar dietary and lifestyle pattern for the duration of the study. The protocol was approved by the Health Sciences Ethical Committee of North-West University (Ethical approval number: NWU-00045-12-S1), conducted in accordance with guidelines for good clinical practice and performed at the Health Clinic of the university. A medical doctor and nurse were present during the entire period of intervention and all cases could leave the premises only after approval by the doctor.

The experiments were conducted on Saturday mornings between 08:00 and 12:00. All subjects had to abstain from breakfast and had to provide an early morning urine sample, collected one hour before the start of the experiment (time –1). The treatments consisted of a measurement of the baseline effect of imbibing the vehicle (500 mL flavored water) that was also used in the remaining three interventions. All the cases were randomly assigned to a treatment/intervention group until all 24 participated in all four interventions. All cases were provided with 1.5 L pure spring water, which was the only substance that could be taken over the period of the experiment. High diurnal variation in urine is well established, and a validated dietary exposure biomarker discovery protocol demonstrated that top-ranked signals discriminating between fasting and 2–4 hour postprandial urine samples could be linked to metabolites abundant between components of a standardized dietary intervention [23]. Thus, urine samples were collected at time zero, just prior to consumption, followed by four further samples at 1, 2, 3 and 4 hours after imbibing the vehicle, providing six samples in total for each case. Owing to commitments of some participants, the experiments were performed over a period of 7 consecutive Saturdays, and all samples were treated and stored as described below.

### Measurement design

Because of the large number of samples produced by this study, their analysis spanned a long time, which necessitated splitting samples across multiple analysis blocks or batches. Analytical assessment of metabolomics data generation proved the highly repeatable nature of NMR data measurements [24]. Although repeatability and reproducibility are not concerns in NMR analyses, the measurement design included the use of pooled quality control (QC) samples to estimate any batch effect or other interfering analytical aspect. Each batch contained the 24 samples (corresponding to 4 treatments across 6 time points) of a single case and three QC samples.

The 27 samples from each batch were analyzed in the following order:

QC_1_ [S_–1_S_0_S_1_S_2_S_3_S_4_]_Vehicle_ [S_–1_S_0_S_1_S_2_S_3_S_4_]_Alcohol_ QC_2_ [S_–1_S_0_S_1_S_2_S_3_S_4_]_NAD_ [S_–1_S_0_S_1_S_2_S_3_S_4_]_NAD+Alcohol_ QC_3_

where S_–1_ represents the sample collected at time –1, S_0_ represents the sample collected at time 0, and so on.

#### Sample collection, characterization and storage

Six samples per intervention were collected from each student. This gave a total of 24 urine samples from each case over the course of the study. One 5 mL and two 1 mL vials were used to provide aliquots of each of the urine samples; these aliquots, together with the remainder of the bulk urine samples, were stored at –80°C. Once all the urine samples were collected, one 1 mL aliquot of each was thawed and combined to prepare a QC sample for the experiment as a whole. This QC sample was then divided into 15 mL aliquots and once again stored at –80°C.

#### Sample preparation and ^1^H-NMR analysis

Spectral analyses were conducted at the NMR facility of the Centre for Human Metabolomics at North-West University. Prior to analysis, an aqueous 1.5 M KH_2_PO_4_ deuterated buffer solution at pH 7.4 was prepared [25]. This solution served to lock the signal during analysis, maintained a stable pH in the sample and contained TSP as the internal standard to provide a chemical shift reference of δ = 0.00. The urine samples, stored at –80°C, were thawed at room temperature for analysis. A 600 μL volume of each sample was centrifuged at 12 000 × g for 5 min to remove any sediments or debris. A 60 μL volume of buffer solution was added to 540 μL of the supernatant, vortexed and transferred to a 5-mm NMR tube.

Each sample so prepared was analyzed on a Bruker Avance III HD 500 MHz NMR spectrometer equipped with a triple-resonance inverse (TXI) ^1^H{^15^N, ^13^C} probe head and x, y, z gradient coils. ^1^H spectra were acquired as 128 transients in 32K data points with a spectral width of 6002 Hz. The sample temperature was maintained at 300 K and the H_2_O resonance was pre-saturated by single-frequency irradiation during a relaxation delay of 4 s, with a 90° excitation pulse fixed at 8 μs. Shimming of the sample was performed automatically on the deuterium signal. The resonance line widths for TSP and metabolites were <1 Hz (measurements at half the height of the peak). Fourier transformation and phase and baseline correction were done automatically. The software used was Bruker Topspin (V3.2) and Bruker AMIX (V3.9.9) [26].

All urine samples were normalized with reference to the creatinine CH_2_ peak at 4.05 ppm. We employed two methods of spectral analysis: (1) the first method consisted of equidistant binning, using a bin width of 0.02 ppm applied to the spectral region of 0.5–10 ppm ((344 bins). This gave a total of 467 integrated units per NMR spectrum, excluding the water region, for each sample for multivariate analysis (S2 File). Based on previous empirical experience with NMR spectral analysis, we defined a threshold value of 2 x 10^6^, being approximately the limit of detection of metabolomic substances presumed to be present in a spectral bin. All values below this threshold were set to zero.

The methodology of processing NMR spectra is well known. Powers [27] describes two schools of thought in this method: peak alignment and binning, the latter of which we used in this study. The equal-binning procedure masks subtle chemical shift differences and hides potentially significant changes of low-intensity peaks, but incurs the risk of splitting peaks or spectral features between bins. The second method used quantified metabolites, derived from variably-sized binning of the original spectral peaks above the noise level. This alternative method prevents peak division between multiple bins, avoiding the problems incurred in the first method. This second approach was specifically applied for the accurate identification and quantification (μmol/mmol creatinine) of discernible metabolites, generating data for univariate analysis.

In addition, we used both the 1D and 2D J-resolved (JRES) approach to further characterize guanidinoacetic acid observed in some urine samples (Section 4 and Figure J in S1 File).

### Data analysis

**Fig 1:**
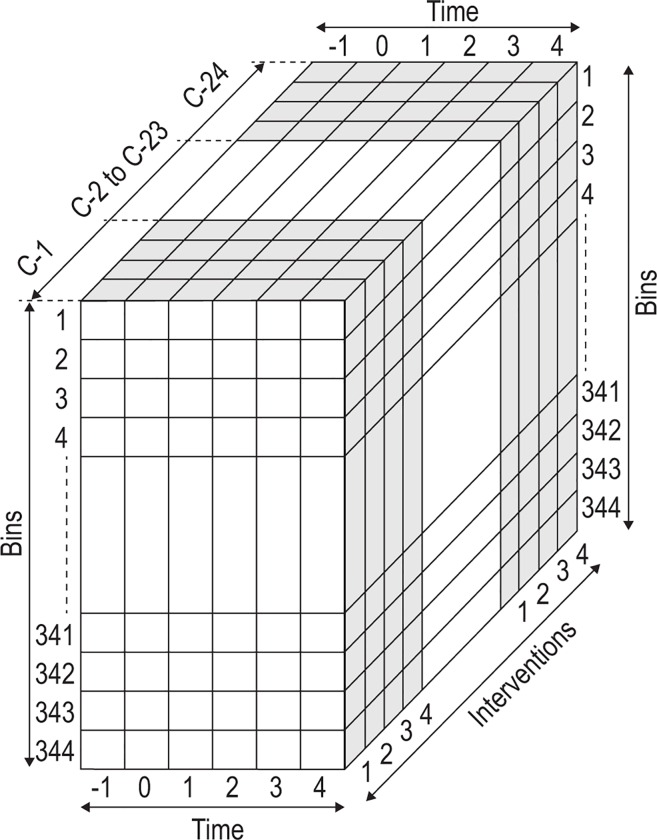
Representation of all elements of the experimental design. The tensor for the complete design consisted of four dimensions: the experimental cases (total 24 –the shaded areas in the figure are for cases 1 and 24), time points (6, hourly), interventions (4, including the vehicle only) and spectral bins (344, following data pretreatment). The total number of 198 144 data points (= 24 x 6 x 4 x 344) thus required bioinformatics analysis to uncover the information from the four interventions. All 24 experimental subjects agreed to participate in each of the four interventions (i.e. a cross-over design), indicated by 1 (vehicle only), 2, 3 and 4. For each of the 24 cases, urine samples (from which the NMR spectral bins were generated) were collected at one hour prior to the intervention (time –1), just before the intervention (time 0) and then at hourly intervals for 4 hours (times 1–4). The results reported in this paper apply to only one of the treatments (consumption of flavored water), which yielded 49 536 data points.

A tensor (Fig 1) was used for three modes of statistical analyses: (1) cross-sectional analysis of time points using traditional multivariate (PCA and PLS-DA) as well as univariate analyses–Wilcoxon signed rank test (WC p-value) and associated fold changes (FC), generated for each bin; (2) ASCA performed across all times; and (3) univariate RM ANOVA for each bin. Further detail on these methods is presented in S1 File.

**Fig 2:**
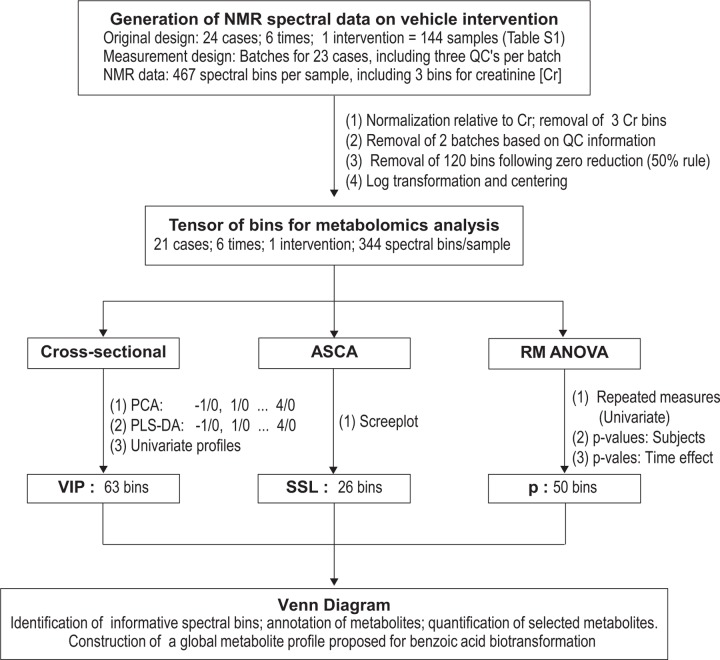
Flow diagram indicating the main lines of activity following data generation, identification and quantification of important metabolites on the intervention towards the proposed biological interpretation. VIP refers to variables important in projection, based on a PLS-DA; SSL refers to the sum of the squared loadings of the ASCA model; p refers to the RM-ANOVA p-values for the time effect.

**Fig 3:**
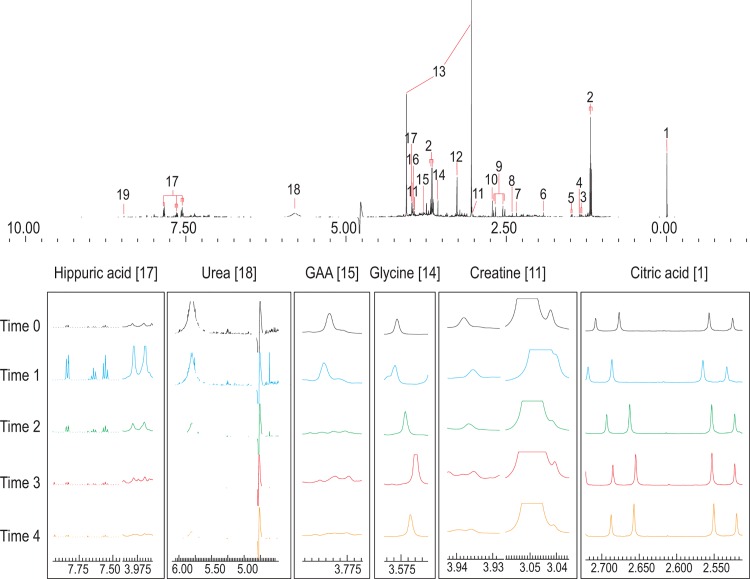
500 MHz ^1^H-NMR spectra of urine. Top spectrum taken from QC sample; numbers indicate the following metabolites: 1: TSP; 2: ethanol; 3: lactic acid; 4: 2-hydroxyisobutyric acid; 5: alanine; 6: acetic acid; 7: pyruvic acid; 8: succinic acid; 9: citric acid; 10: dimethylamine; 11: creatine; 12: trimethylamine-N-oxide (TMAO); 13: creatinine; 14: glycine; 15: guanidinoacetic acid (GAA); 16: glycolic acid; 17: hippuric acid; 18: urea; 19: formic acid. The six important metabolites (high VIP values) are highlighted below to qualitatively show differences over time 0 (black), 1 (blue), 2 (green), 3 (red) and 4 (orange)–scaled according to creatinine. Apart from citric acid and hippuric acid, the detoxification product of benzoic acid, no other constituents from the vehicle appeared to be detectable in the NMR analysis.

The original spectral data, derived from the cases following the vehicle intervention, sampling at six time points and including the QCs (Section 1 and Table A in S1 File), yielded a total of 144 samples, as shown in the flow diagram (Fig 2) and illustrated through a representative NMR spectrum for one QC sample (Fig 3). However, during the experiment one participant did not complete all four interventions, thus yielding 138 samples obtained from the remaining 23 cases, which were used in the data analysis. The original spectral data were pre-processed by normalization relative to the creatinine content (between 4.05 and 4.07 ppm); replacing very low values with zero and performing a 50% zero-filter. Case reduction was based on batch comparisons of the QC samples (detail presented in S1 File)–two batches were found to be outliers and were removed.

A list of important bins was generated by each of the three modes and combined using a Venn diagram approach to identify a final shortlist of important spectral bins. This shortlist contained information on the main metabolites that reflected the intervention.

## Results

### Cross-sectional analysis

One student did not complete the full intervention program, and was thus removed from the group. All results were finally based on the 344 ^1^H-NMR profiled bins for 21 cases, following the elimination of two outlier batches (i.e. data from two cases), across the 6 time points. General time-dependent changes in the spectral data were first evaluated with regard to the baseline, i.e. time 0. Cross-sectional comparisons of time points –1, 1, 2, 3 and 4 hours vs. time 0 were performed using three multivariate approaches (unsupervised Euclidian and Ward hierarchical cluster analyses presented as dendrograms, unsupervised PCA and supervised PLS-DA models) and the combination of two univariate approaches (the p-values from Wilcoxon signed rank test and fold change (FC) values presented as Volcano plots). The data were log transformed (shifted natural logarithm with shift parameter set to 1) and centered prior to performing PCA and PLS-DA. The remaining methods were applied to untransformed data. The statistical techniques used and the results of all time points for the cluster analyses, Volcano plots, as well as for the PCA and PLS-DA, are discussed in detail in S1 File and Matlab coding given as supplementary file (S3 File).

**Fig 4:**
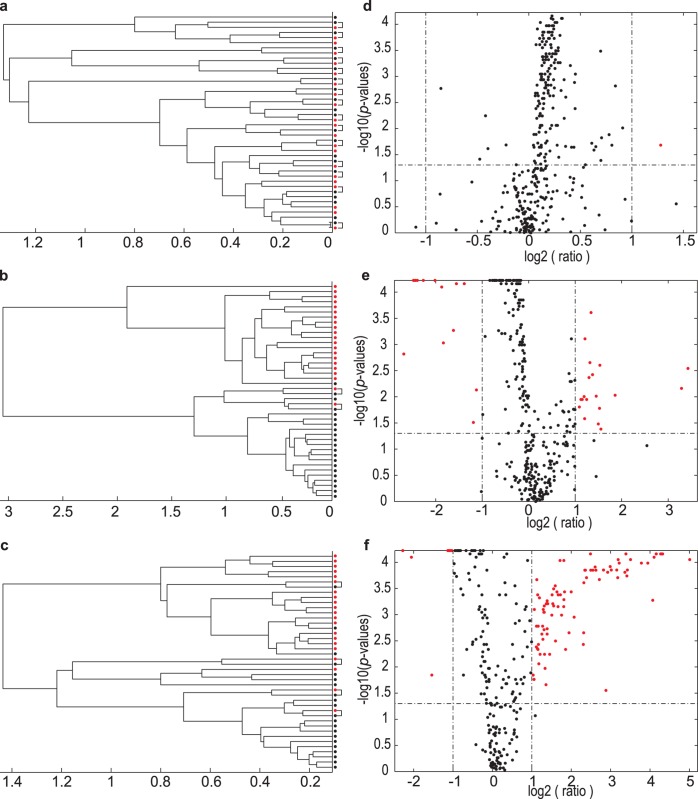
Group separation among experimental groups through dendrograms and Volcano plots based on equidistant binning spectral data. (**a**–**c**) Dendrograms from cluster analysis of 21 cases are shown. The dendrograms were constructed based on subsets of the data representing time 0 along with the data obtained one hour before the intervention (time –1); one hour; and four hours following the intervention (times 1 and 4), respectively. Data for all cases at time 0 are shown as black dots and at the other time slots as red dots. Time points from the same individuals that clustered close together are indicated by squared brackets, linking them. (**d**–**f**) Volcano plots for the same time points as in **a**–**c** indicate the distribution of the individual bins, based on FC and WC p-values. Demarcation of important bins is shown by the horizontal and vertical dotted lines. The number of influential bins is shown as red dots in the rectangles of the upper left and right segments of each Volcano plot.

Fig 4 indicates the data from time –1 hour, 1 hour and 4 hours relative to time 0 for the cluster analysis and Volcano plots.

Fig 4A shows little separation (time –1 relative to time 0) between the individual cases, indicating a perceived closeness of spectral data encapsulated in the NMR bins for the individual cases. The metabolite profiles detected in the urine of most samples collected immediately prior to consumption of the vehicle thus represent the profile following 12 hours fasting. This conclusion also holds for the case indicated as an outlier bin within the group as a whole (indicated by a red dot in Fig 4D). The distinct group separation after vehicle consumption (Fig 4B and 4C) clearly indicates a metabolic perturbation. The results for the other two multivariate methods (PCA: Figure D and PLS-DA: Figure E in S1 File) confirmed the main observations shown in the dendrograms: no group separation between the data obtained at time zero and one hour before the intervention (Figures D(A) and E(A) in S1 File), clear separation one hour after consumption, i.e. time 0 vs. time 1 (Figures D(B) and E(B) in S1 File). Characteristic of multivariate methods, the group separations were more evident when applying a supervised (PLS-DA) as opposed to an unsupervised method (PCA). The Volcano plots revealed that a single bin indicated a significant (p ≤ 0.05) up-regulated value (FC ≥ 2.0) at time 0 with respect to time –1 (Fig 4D), which increased to a total of 31 up- and down-regulated values (Fig 4E) one hour after the intervention (p ≤ 0.05 and |FC| ≥ 2) and becoming abundant after 4 hours (Fig 4F), as compared to the baseline measurement (time 0).

### Inter-individual variation following vehicle consumption

**Fig 5:**
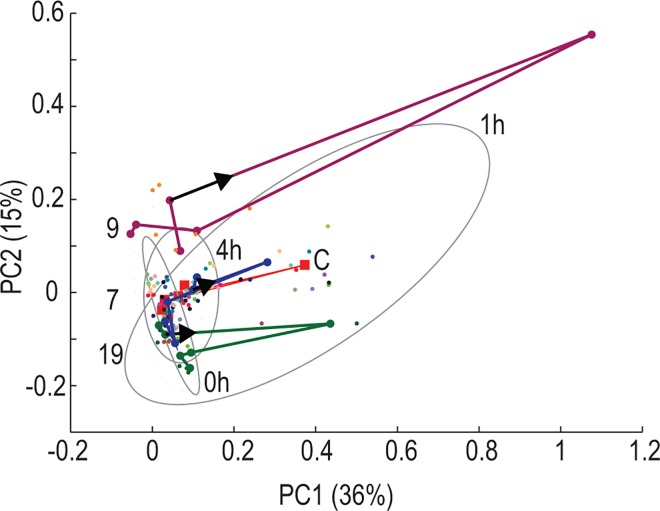
Unfolded PCA Scores Plots. PC1 and PC2 for all individual cases (colored dots) and indication of the 95% confidence ellipsoids for scores of PC1 and PC2 for times –1, 1 and 4 hours. The centroids for these three clusters are indicated as red squares. The trajectories of the spectral profiles of three individuals are also shown. The trajectories of three cases (numbers 9, 7 and 19 are shown as purple, blue and green lines, respectively) illustrate the inter-individual responses to the intervention. The direction of the trajectories is indicated by the short black arrow, starting from time 0, for clarity.

Fig 5 represents the scores based on the first two principal components (PC1 and PC2) of the PCA model for the unfolded data tensor. The data tensor was unfolded in time as described and illustrated in S1 File [21]. The unfolding transforms a three-dimensional tensor into a two-dimensional matrix and thus allows for PCA.

PCA of the unfolded tensor provides insight into the effect of the vehicle in time on the bins (indicated by the ellipses and centroids in Fig 5) as well as for individual cases, shown by the dots and trajectories in Fig 5. It is evident from Fig 5 that there is a change in the global profile (i.e. across all cases) over time. Times –1, 0, 2, 3 and 4 appear to be quite similar, with a dramatic change visible after 1 hour (time 1). It was evident from the prior analyses (Figs 4 and 5) that the urinary spectral profiles following the consumption of flavored water suggested notable changes in the metabolite profiles of all individuals in response to the intervention. To illustrate the inter-individual variation, the centroids of the PCA for the group (all 21 cases) and individual cases were tracked and highlighted over the period from time 0 to time 4 hours (Fig 5). The trajectories drawn in Fig 5 represent only three selected individuals, for clarity. The selected trajectories (as well as for the group as a whole, Fig 5) indicate clear similarities in the individual responses to vehicle consumption in time: the profiles of the trajectory from time 0 to 4 hours were comparable in some respects. However, distinct differences were also noted: the response at time 1 hour following vehicle consumption in case 9 gives the impression of being an outlier. However, since the QC samples for this individual (linked to a given batch) were not outliers, we attribute this variation to the unique response of the case to the vehicle. Since we are interested in these kinds of responses, the observation was retained. Overall, the observations shown in Fig 5 indicate that the cases themselves are a noteworthy source of variation.

### Longitudinal response to vehicle consumption

**Fig 6:**
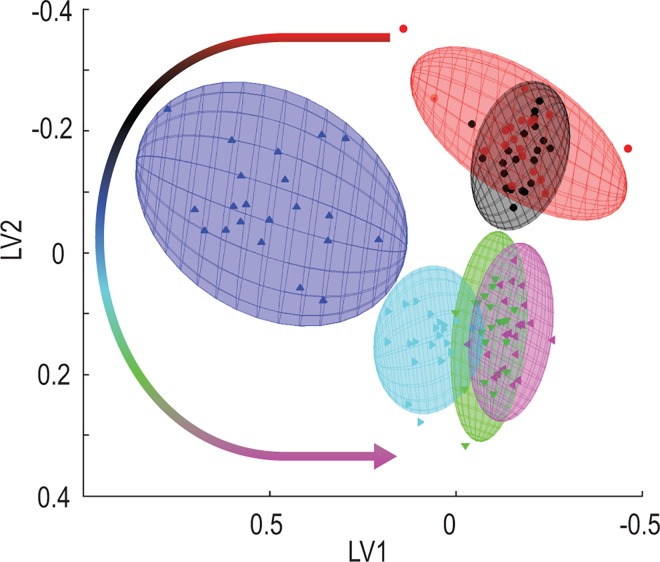
Application of ASCA to the 344 NMR spectral bins for 21 individuals over the period of the intervention. Plots of the sum of the ASCA effects and projected residuals are color coded according to time following the intervention (–1, 0, 1, 2, 3 and 4 hours shown in red, black, blue, light blue, green and pink, respectively), with the arrow showing the time-dependent trend, using the same discriminating color sequence.

The results presented thus far suggest that the variation in the total data set was based on the intervention and superimposed by the normal inter-individual heterogeneity and time effects and by their interactions. For optimal insight into the effect of the intervention, and specifically to identify the most important metabolites reflecting the metabolic perturbation induced by the flavored water, we used three complementary approaches. The traditional PLS-DA (illustrated in Figure E in S1 File) was used to find an approximation of the biological profile following the intervention. We identified 63 bins as important based on a maximum VIP over the five comparisons greater than 2. Second, ASCA (Fig 6) was applied as it provided an approach which was developed to deal with multivariate data sets based on a defined experimental design, including time-dependent data [4]. Again, the data were log transformed and centered. Similar to the unfolded PCA result, the plot of the sum of the ASCA effects and projected residuals onto the first two components of the effects subspace [28], shown in Fig 6, indicate that observations at times –1, 0 are quite similar, indicating a state of homeostasis that existed after the fasting period. A marked change became visible one hour after the intervention. This was followed by comparable profiles, especially between times 3 and 4, indicating a return to a state of homeostasis, which was not identical to the state prior to the intervention, as will be discussed below. The sum of the squared loadings (SSL) of the ASCA model were ranked in decreasing order and used as a scree plot (Figure G in S1 File) to identify 26 bins with notably higher SSL values.

Third, RM ANOVA was used to assess each bin individually to identify bins that changed significantly in time, from which we selected the top 50 bins (p-values less than 0.00001) which appeared to be most informative. Data, generated from complex experimental designs such as the intervention study presented here, produce information that intersect- in various ways.

### Identification of important variables

**Fig 7:**
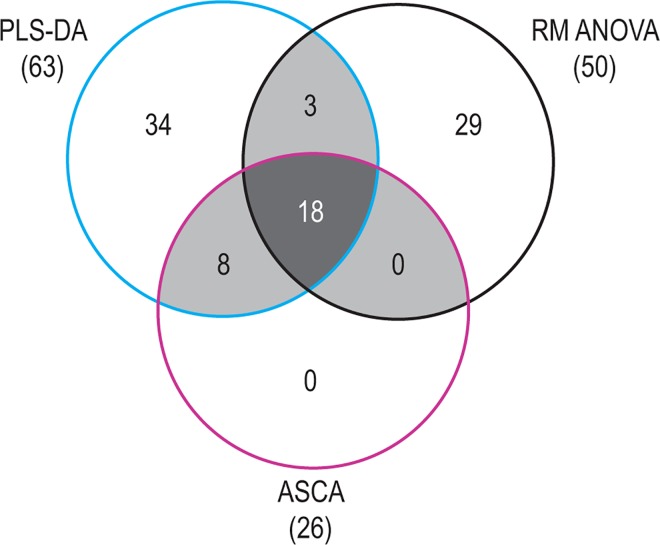
Venn diagram. Bins which are common to any two statistical approaches are indicated by the lightly shaded areas, and the 18 bins that are common to all three are shown in the central, heavily shaded area.

A Venn diagram (Fig 7) was used to visualize the counts (number of bins) of the lists of important bins sharing some properties. Three properties were selected: maximum VIP ≥ 2 from the PLS-DA, SSL > 0.01 from the ASCA, and p-values from the RM ANOVA as defined above. A total of 29 bins were shortlisted, derived from being present in at least two of the three lists and which were unique in the intersection reflected by the shaded areas in Fig 7. It is interesting to note the power of the ASCA approach as it was able to identify 26 of the 29 bins, but this does not imply that variables not selected (e.g. the 29 RM ANOVA metabolites) are all unimportant as each method selects variables in its own way. For our purpose we concentrated, however, on the shared metabolites; how this was achieved is described in more detail in Section 3.6 of S1 File. The list of 29 bins provided the final selection of bins to be identified as important metabolites and quantified to concentrations. Detailed information on the application of ASCA, RM ANOVA and unfolded PCA is presented in Section 3 of S1 File.

Analysis of the spectral characteristics of the 29 bins indicated that 23 bins related to endogenous human metabolites. Spectral information from four bins could not be assigned to any known chemical substance and two bins indicated exogenous contaminants. Some bins (indicated in brackets) had structural information on the same metabolite, which resulted in a list of six key metabolites: urea (10 bins), hippuric acid (8 bins) and citric acid (2 bins) as well as creatine, 3-methylhistidine and guanidinoacetic acid each with one bin. One bin for each of the six metabolites, as well as for glycine (not identified as a highly perturbed metabolite), were quantified for all time points, as summarized in Table 1.

**Table 1:** Quantified data on metabolically important metabolites. All quantified values included are from NMR-determined urine analyses; the references are all from the Human Metabolome Database, and are expressed as μmoles metabolite/mmole creatinine. WC p-values are based on the comparison of the respective metabolite concentrations from time = 1 hour to 4 hours, relative to time zero.

Information on important metabolites		Time-dependent characteristics of important metabolites following flavored water consumption								
Metabolite	Normal values	Mean	Mean	WC	Mean	WC	Mean	WC	Mean	WC
(δ [ppm], multiplicity and chemical group)	Mean [Range]	[SD]	[SD]	p-value	[SD]	p-value	[SD]	p-value	[SD]	p-value
	[Reference]	Time:0	Time:1		Time:2		Time:3		Time: 4	
Citric acid	203[49–600]	213	264	˂0.0005	322	˂0.0005	320	0.001	306	0.002
(2.59 AB—[(CH_2_)_2_])	[35]	[115]	[137]		[139]		[126]		[117]	
Hippuric acid	217[28–610]	210	884	˂0.0005	449	˂0.0005	285	0.021	228	0.689
(3.97d - [CH_2_])	[35]	[100]	[268]		[170]		[154]		[142]	
Glycine	106[44–300]	94	114	0.001	117	0.005	115	0.014	108	0.122
(3.57 s—[CH_2_])	[35]	[63]	[58]		[37]		[34]		[32]	
Guanidinoacetic acid	89[11–124]	97	77	0.173	0	0.028	0	0.028	0	0.028
(3.78 s—[CH_2_])	[36]	[193]	[200]		0		0		0	
Urea	12285[174–49097]	479	751	˂0.0005	1004	˂0.0005	937	˂0.0005	914	˂0.0005
(5.78 broad—[(NH_2_)_2_])	[35]	[230]	[322]		[303]		[311]		[291]	
Creatine	46[3–448]	44	57	0.015	37	0.079	35	0.011	33	0.003
(3.04 s—[CH_3_])	[35]	[20]	[27]		[14]		[9]		[7]	

Excretion kinetics for all six these metabolites are shown in S1 File (Figures H(A)–H(F)). The most conspicuous change–indicating the highly efficient detoxification of benzoic acid–occurred in the more than fourfold (p < 0.0005) increased urinary excretion of hippuric acid within one hour of consuming the flavored water, and its return to the same level as before the intervention (Table 1 and Figure H(A) in S1 File). As indicated by the unfolded PCA scores (Fig 5), the trajectories of individual cases (illustrated for cases 7, 9 and 19) tended to return to positions close, but not identical, to the pre-intervention positions. The excretion kinetics of the six metabolites (Figure H in S1 File) and the Wilcoxon signed rank p-values (Table 1) gave strong numerical indications for this observation: (1) Hippuric acid dominated the profiles, and returned to practically the same state as prior to the intervention (p = 0.689, Time 0 vs Time 4). (2) The levels of guanidinoacetic acid (p = 0.028) and creatine (0.003) were significantly lower than prior to the intervention (Time 0 vs Time 4). (3) The excretion of citric acid (p = 0.002) and urea (p<0.0005) were still after four hours significantly higher than prior to the intervention (Time 0 vs Time 4). Moreover, the excretion profiles of some unknown and unimportant metabolites, gave further support for the observed trajectory profiles (Figure I in S1 File).

The interpretation of changes in the excretion of these remaining metabolites paved the way for the construction of a metabolic profile, reflecting the consequences of detoxification of a single xenobiotic–benzoic acid as used here–as discussed below.

## Discussion

**Fig 8:**
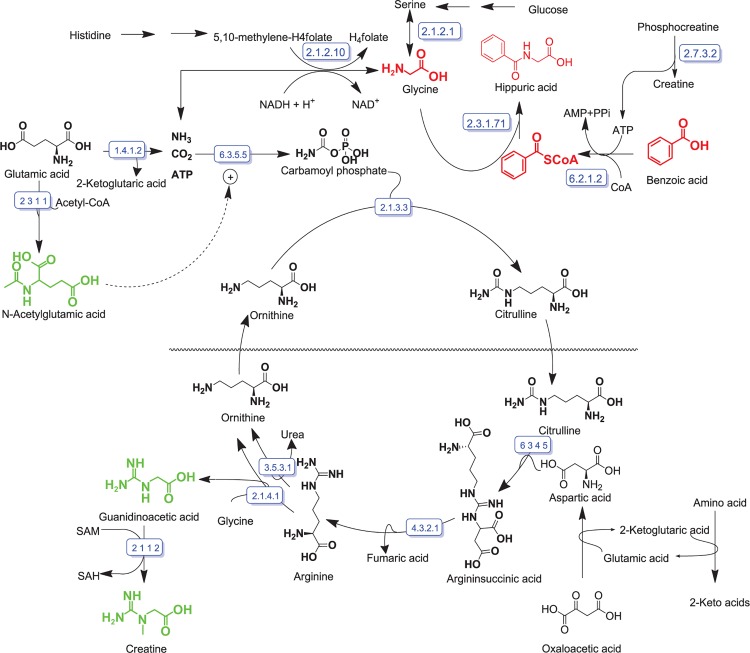
Metabolite profile of benzoic acid biotransformation with hippuric acid as outcome. The metabolites of the primary biotransformation reactions are shown in red. Metabolites of metabolic reactions proposed as being associated with the biotransformation reactions are shown in black. [Metabolites shown in green are presumed to decrease due to increased glycine demand and synthesis of glycine through the reversed glycine cleavage system.] Enzyme nomenclature (names accepted by the IUBMB): EC 1.4.1.2: glutamate dehydrogenase; EC 2.1.1.2: guanidinoacetate N-methyltransferase; EC 2.1.2.1: glycine hydroxymethyltransferase (alt.: serine hydroxymethyltransferase); EC 2.1.2.10: aminomethyltransferase (a glycine synthetase); EC 2.1.3.3: ornithine carbamoyltransferase; EC 2.1.4.1: glycine amidinotransferase; EC 2.3.1.1: amino-acid N-acetyltransferase; EC 2.3.1.71: glycine N-benzoyltransferase (a GLYAT); EC 2.7.3.2: creatine kinase; EC 3.5.3.1: arginase; EC 4.3.2.1: argininosuccinate lyase; EC 6.2.1.2: butyrate-CoA ligase (a medium-chain acyl-CoA ligase); EC 6.3.4.5: argininosuccinate synthase; EC 6.3.5.5: carbamoyl-phosphate synthase (glutamine-hydrolyzing)

Using the combination of important metabolites identified employing the three statistical methods (Fig 7), the quantified information (Table 1) and biochemical interpretation of the metabolomics data, we constructed a metabolite profile reflecting the primary GLYAT-catalyzed biotransformation of benzoic acid and the metabolic consequences of benzoic acid ingested via flavored water (Fig 8).

Humans conjugate a variety of aliphatic and aromatic monocarboxylic acids with several amino acids; the resulting peptides are excreted in the urine and bile. In characterizing two closely related GLYATs, Nandi *et al*. [18] indicated through kinetic studies that benzoyl-CoA is the main substrate for a benzoyltransferase, with salicyl-CoA and certain aliphatic acyl-CoAs being lesser substrates for it as well. The closely related phenylacetyltransferase utilize phenylacetyl- and indoleacetyl-CoA as substrates. Acyl-CoA substrates of one transferase did not serve as substrate for the other, but act as competitive inhibitors. Glycine is the preferred acyl acceptor for both enzymes, with K_m_^App^ for benzoyltransferase being less (3 mM glycine) than for the phenylacetyltransferase (20 mM glycine). Due to the high activity of glutamine N-phenylacetyltransferase, phenylacetylglycine is almost never detected in human urine. Almost all the phenylacetic acid is excreted as N-phenylacetylglutamine. It thus may be anticipated that formation of hippuric acid in this intervention study mainly reflects the GLYAT activity of the benzoyl variant (EC 2.3.1.71).

Early studies showed that rat liver mitochondria synthesize hippuric acid at a rate of up to 4 nmol/min per mg of protein [29] and comparative kinetic analysis suggested that the formation of the benzoyl-CoA substrate is the rate-limiting factor. More recently, two distinct forms of xenobiotic/medium-chain fatty acid:CoA ligase (XM-ligase) were isolated from human liver mitochondria, referred to as HXM-A and HXM-B [30]. Both forms had medium-chain fatty acid:CoA ligase activity but HMX-A showed 60–80% activity towards 15 different carboxylic acids relative to benzoic acid, its best xenobiotic substrate (100% activity and the highest V_max_/K_m_). Hexanoic acid was the best substrate for HXM-B, although it was also active towards xenobiotic carboxylic acids. In accordance with these findings we propose that medium-chain acyl-CoA ligase (EC 6.2.1.2) is a major catalyst for production of the CoA-substrate required for hippuric acid formation in the present study.

Acyl-CoA ligases/synthetases belong to a superfamily of adenylate-forming enzymes, and catalyze the two-step activation of fatty acids or carboxylate-containing xenobiotics [31], with xenobiotic-CoA formation in parallel to endogenous fatty acid activation through the role of the ATP-dependent acid:CoA ligases [32]. We visualize the activation of benzoyl-CoA through a Bi Uni Uni Bi Ping-Pong molecular mechanism proposed for CoA-substrate formation by a long-chain fatty acyl-CoA synthetase [33]: The benzoyl carboxylate substrate (BA) first reacts with enzyme-bound ATP to form an acyl-adenylate intermediate (Ec:B~AMP), which then reacts with CoA to produce the activated benzoyl-CoA ester (B~CoA), having pyrophosphate (PPi) and AMP as byproducts.

Formation of hippuric acid, following GLYAT-catalyzed conjugation between benzoyl-CoA and glycine, peaks within one hour (p < 0.0001) following the consumption of flavored water (Table 1). The p-values of the Wilcoxon signed rank test (Table 1) for the time-dependent urinary excretion of glycine complements that of hippuric acid, and likewise peaks at one hour (p = 0.001) following the intervention. However, the excretion kinetics profile of glycine clearly suggests abundance of glycine during the main detoxification period (two hours after the intervention), rather than glycine depletion. Two main pathways exist for increased glycine biosynthesis (Fig 8) under physiological conditions of glycine demand: (1) L-serine can be converted to glycine by serine hydroxymethyltransferase (EC 2.1.2.1) in the reversible glycine biosynthesis pathway, having tetrahydrofolate as acceptor for the CH_2_OH group from serine, yielding 5,10-methylene-tetrahydrofolate and water. (2) The *de novo* synthesis of glycine can occur from CO_2_ and NH_3_, catalyzed by glycine synthase (EC 2.1.2.10) using 5,10-methylene-tetrahydrofolate as the source of the second carbon and yielding tetrahydrofolate–likewise in a reversible reaction. The CO_2_ and NH_3_ substrates for the *de novo* synthesis of glycine are generated by mitochondrial glutamate dehydrogenase (EC 1.4.1.2) that converts glutamic acid to 2-ketoglutaric acid, with NH_3_ usually as a substrate in the urea cycle. Glycine is also a substrate for glycine transamidinase (EC 2.1.4.1)-catalyzed synthesis of guanidinoacetic acid. Urinary guanidinoacetic acid concentrations, notably, decreased (Table 1; p = 0.173) within the first hour following consumption of flavored water, towards values below the detection limit (Table 1; p = 0.028) two hours later. We speculate that decreased guanidinoacetic acid is caused by the preferential utilization of glycine for benzoic acid detoxification as well as lower urea cycle activity due to decreased N-acetylglutamic acid, a modulator for the up-regulation of the urea cycle. Against this background it seems that the traditional paradigm of GLYAT-catalyzed benzoic acid detoxification, supported by increased *de novo* glycine biosynthesis (Fig 8), prevails under the conditions of the present intervention experiment, even though up-regulation of glycine-amidinotransferase (EC 2.1.4.1) through decreased creatine [34] cannot be excluded.

Finally, we observed an increase in urinary creatine within the first hour following the intervention. The creatine/phosphocreatine system plays an important role in energy storage and energy provision, with creatine synthesis being central in cellular energy metabolism. Two main enzymes are the basis of the creatine biosynthesis pathway, namely, arginine:glycine amidinotransferase (EC 2.1.4.1) and S-adenosyl-L-methionine:N-guanidinoacetate methyltransferase (EC 2.1.1.2), as shown in Fig 8. Given the decrease of guanidinoacetic acid to below the detection limit (Table 1), it seems unlikely that the urinary creatine originates from, and excess creatine is produced by, the creatine biosynthesis pathway. Our proposal is that phosphocreatine (EC 2.7.3.2) degrades in favor of creatine and supplements the ATP reserves, required for the burst of ATP required following benzoic acid detoxification.

In summary, we have described the metabolite profile following benzoic acid intake as part of a designed intervention study. The time-dependent glycine profile supported the view of abundant glycine availability during the main detoxification period rather than that of glycine depletion. It should be noted, however, that the perturbation caused by benzoic acid consumption may be more complex than discussed above. We observed small, but significant time-dependent changes in the NMR spectra (Figure I in S1 File) for methylguanidine and three unknown substances that are not accounted for in the metabolic model shown in Fig 8.

The complex NMR spectral data, generated from cases participating in a time-dependent cross-over study, could be resolved sufficiently through the application of traditional univariate and multivariate analyses combined with an ANOVA-simultaneous component analysis (ASCA), repeated measures analysis of variance (RM ANOVA) and unfolded principal component analysis (unfolded PCA)–an approach that opens a novel way for analyses and understanding of complex metabolomics data that reflect perturbations from normal or pathophysiological endogenous or exogenous origin. Furthermore the combination of the complementary statistical methods together with the transdisciplinary approach followed in metabolomics research provided a more holistic view of the data. This proved useful in elucidating the effect of the intervention and provided novel insights information into benzoic acid biotransformation, which is not typically observed by more reductionist methods of analysis.

## Supporting Information

S1 File (DOCX)

S2 File (XLSX)

S3 File (M)

## References

[1] Gibney MJ, Walsh M, Brennan L, Roche HM, German B, Van Ommen B. Metabolomics in human nutrition: opportunities and challenges. Am J Clin Nutr. 2005; 82:497–503. 16155259 10.1093/ajcn.82.3.497

[2] Wishart DS. Metabolomics: applications to food science and nutrition research. Trends Food Sci Tech. 2008; 19:482–493.

[3] Antti H, Bollard ME, Ebbels T, Keun H, Lindon JC, Nicholson JK, et al Batch statistical processing of ^1^NMR-derived urinary spectral data. J Chemometrics. 2002; 16:461–468.

[4] Smilde AK, Jansen JJ, Hoefsloot HCJ, Lamers R-JAN., Van der Greef J, Timmerman ME. ANOVA-simultaneous component analysis (ASCA): a new tool for analyzing designed metabolomics data. Bioinformatics. 2005; 21:3043–3048. 10.1093/bioinformatics/bti476 15890747

[5] Park H-W, Park EH, Yun H-M, Rhim H. Sodium benzoate mediated cytotoxicity in mammalian cells. J Food Biochem. 2011; 35:1034–1046.

[6] Schachter D, Taggart JV. Benzoyl-coenzyme A and hippurate synthesis. J Biol Chem. 1953; 203:925–934. 13084662

[7] Conti A, Bickel MH. History of drug metabolism: discoveries of the major pathways in the 19th century. Drug Metab Rev. 1977; 6:1–50.608410

[8] Beyoğlu D, Smith RL, Idle JR. Dog bites man or man bites dog? The enigma of the amino acid conjugations. Biochem Pharmacol. 2011; 83:1331–1339. 10.1016/j.bcp.2011.12.031 22227274 PMC3314100

[9] Beyoğlu D, Idle JR. The glycine deportation system and its pharmacological consequences. Pharmacol Therapeut. 2012; 135:151–167. 10.1016/j.pharmthera.2012.05.003 PMC3665358 22584143

[10] Wen H, Yang H-J, An YJ, Kim JM, Lee DH, Jin X, Park S-W, Min K-J, Park S. Enhanced phase II detoxification contributes to beneficial effects of dietary restriction as revealed by multi-platform metabolomics studies. Mol Cell Proteomics. 2013; 12:575–586. 10.1074/mcp.M112.021352 23230277 PMC3591652

[11] Nicholson JK, Holmes E, Wilson ID. Gut microorganisms, mammalian metabolism and personalized health care. Nat Rev Microbiol. 2005; 3:431–438. 10.1038/nrmicro1152 15821725

[12] Jana S, Mandlekar S. Role of phase II drug metabolizing enzymes in cancer chemoprevention. Curr Drug Metab. 2009; 10:595–616. 19702535 10.2174/138920009789375379

[13] Zimniak P. Detoxification reactions: relevance to aging. Ageing Res Rev. 2008; 7:281–300. 10.1016/j.arr.2008.04.001 18547875 PMC2671233

[14] Holtzclaw WD, Dinkova-Kostova AT, Talalay P. Protection against electrophile and oxidative stress by induction of phase 2 genes: the quest for the elusive sensor that responds to inducers. Adv Enzyme Regul. 2004; 44:335–367. 10.1016/j.advenzreg.2003.11.013 15581500

[15] Aprison MH, Werman R. The distribution of glycine in cat spinal cord and roots. Life Sci. 1965; 4:2075–2083. 5866625 10.1016/0024-3205(65)90325-5

[16] Tanaka I, Ezure K. Overall distribution of GLYAT2 mRNA-containing versus GAD67 mRNA-containing neurons and colocalization of both mRNAs in midbrain, pons, and cerebellum in rats. Neurosci Res. 2004; 49:1874–1882. 10.1016/j.neures.2004.02.007 15140559

[17] Badenhorst CPS, Van der Sluis R, Erasmus E, Van Dijk AA. Glycine conjugation: importance in metabolism, the role of glycine N-acyltransferase, and factors that influence interindividual variation. Expert Opin Drug Met. 2013; 9:1139–1153. 10.1517/17425255.2013.796929 23650932

[18] Nandi DL, Lucas SV, Webster LT Jr. Benzoyl-Coenzyme A:Glycine N-acyl-transferase and Phenylacetyl-Coenzyme A:Glycine N-Acyltransferase from Bovine Liver Mitochondria. J Biol Chem. 1979; 254:7230–7237. 457678

[19] Schachter D, Taggart JV. Glycine N-acylase: purification and properties. J Biol Chem. 1954; 208:263–275. 13174534

[20] Lindon JC, Holmes E, Nicholson JK. Pattern recognition methods and applications in biomedical magnetic resonance. Prog Nucl Magn Reson Spectrosc. 2001; 39:1–40.

[21] Villez K, Steppe K, De Pauw DJW. Use of unfold PCA for on-line plant stress monitoring and sensor failure detection. Biosystems Engineering. 2009; 103:23–24.

[22] Mason S. The metabolomics of acute alcohol abuse. M.Sc. Thesis, North-West University. 2010. Available: http://dspace.nwu.ac.za/handle/10394/5034

[23] Favé G, Beckmann M, Lloyd AJ, Zhou S, Harold G, Lin W, et al Development and validation of a standardized protocol to monitor human dietary exposure by metabolite fingerprinting of urine samples. Metabolomics. 2011; 7:469–484. 10.1007/s11306-011-0289-0 22039364 PMC3193537

[24] Viant M, Bearden DW, Bundy JG, Burton IW, Colette TW, Ekman DR, et al International NMR-based environmental metabolomics intercomparison exercise. Environ Sci Technol. 2009; 43:219–225. 19209610 10.1021/es802198z

[25] Dona AC, Jiménez B, Schaäfer H, Humpfer E, Spraul M, Lewis MR, et al Precision high-throughput proton NMR spectroscopy of human urine, serum, and plasma for large-scale metabolic phenotyping. Anal Chem. 2014; 86(19):9887–9894. 10.1021/ac5025039 25180432

[26] Ellinger JJ, Chylla RA, Ulrich EL, Markley JL. Databases and Software for NMR-based metabolomics. Curr Metabolomics. 2013; 1:28–40. 10.2174/2213235X11301010028 PMC3832261 24260723

[27] Powers R. NMR Metabolomics and drug discovery. Magn Reson Chem. 2009; 47, S2–S11. 10.1002/mrc.2461 19504464

[28] Zwanenburg G, Hoefsloot HCJ, Westerhuis JA, Jansen JJ, Smilde AK. ANOVA–principal component analysis and ANOVA–simultaneous component analysis: a comparison. J Chemometrics. 2011; 25:561–567.

[29] Gatley SJ, Sherratt HAS. The synthesis of hippurate from benzoate and glycine by rat liver mitochondria. Biochem J. 1977; 166:39–47. 901416 10.1042/bj1660039 PMC1164954

[30] Vessey DA, Kelley M, Warren RS. Characterization of the CoA ligases of human liver mitochondria catalyzing the activation of short and medium-chain fatty acids and xenobiotic carboxylic acids. Biochim Biophys Acta. 1999; 1428:455–462. 10434065 10.1016/s0304-4165(99)00088-4

[31] Kochan G, Pilka ES, von Delft F, Oppermann U, Yue WW. Structural snapshots for the conformation-dependent catalysis by human medium-chain acyl-coenzyme A synthetase ACSM2A. J Mol Biol. 2009; 388:997–1008. 10.1016/j.jmb.2009.03.064 19345228

[32] Knights KM, Drogemuller CJ. Xenobiotic-CoA Ligases: Kinetic and Molecular Characterization. Curr Drug Metab. 2000; 1:49–66. 11467080 10.2174/1389200003339261

[33] Hisanaga Y, Ago H, Nakagawa N, Hamada K, Ida K, Yamamoto M, et al Structural basis of the substrate-specific two-step catalysis of long chain fatty acyl-CoA synthetase dimer. J Biol Chem. 2004; 279:31717–31726. 10.1074/jbc.M400100200 15145952

[34] McGuire DM, Gross MD, Van Pilsum JF, Towle HC. Repression of rat kidney L-arginine:glycine amidinotransferase synthesis by creatine at a pretranslational level J Biol Chem. 1984; 259:12034–12038. 6384218

[35] Bouatra S, Aziat F, Mandal R, Guo AC, Wilson MR, Knox C, Bjorndahl TC, Krishnamurthy R, Saleem F, Liu P, Dame ZT, Poelzer J, Huynh J, Yallou FS, Psychogios N, Dong E, Bogumil R, Roehring C, Wishart DS. The human urine metabolome. PLOS ONE. 2013; 8(9):e73076 10.1371/journal.pone.0073076 24023812 PMC3762851

[36] Fingerhut R. Stable isotope dilution method for the determination of guanidinoacetic acid by gas chromatography/mass spectrometry. Rapid Commun Mass Spectrom. 2003;17(7):717–722 10.1002/rcm.966 12661026

