# Supplementary material for: Contribution towards a Metabolite Profile of the Detoxification of Benzoic Acid through Glycine Conjugation: An Intervention Study
Source: PLoS One. 2016 Dec 1;11(12):e0167309. doi: 10.1371/journal.pone.0167309 (PMC5132330; doi:10.1371/journal.pone.0167309)
Supplement: S1 File — (DOCX) [file pone.0167309.s001.docx]

**Supporting Information**

**Contribution towards a Metabolite Profile of the Detoxification of Benzoic Acid through Glycine Conjugation: An Intervention Study**

Cindy Irwin^1,¶^, Mari van Reenen^1,2,¶^, Shayne Mason^1^, Lodewyk J. Mienie^1^, Johan A. Westerhuis^2,3^ and Carolus J. Reinecke^1,*^

^1^Centre for Human Metabolomics, Faculty of Natural Sciences, North-West University (Potchefstroom Campus), Potchefstroom, South Africa

^2^Department of Statistics, Faculty of Natural Sciences, North-West University (Potchefstroom Campus), Potchefstroom, South Africa

^3^Biosystems Data Analysis, Swammerdam Institute for Life Sciences, University of Amsterdam, Amsterdam, The Netherlands

* Corresponding author

E-mail: [carools.reinecke@nwu.ac.za](mailto:carools.reinecke@nwu.ac.za)

^¶^ These authors contributed equally to this work

**Email addresses:**

Cindy Irwin: [cindyirwin74@gmail.com](mailto:cindyirwin74@gmail.com)

Mari van Reenen: [12791733@nwu.ac.za](mailto:12791733@nwu.ac.za)

Shayne Mason: [nmr.nwu@gmail.com](mailto:nmr.nwu@gmail.com)

Lodewyk J. Mienie: [japie.mienie@gmail.com](mailto:japie.mienie@gmail.com)

Johan A. Westerhuis: [J.A.Westerhuis@uva.nl](mailto:J.A.Westerhuis@uva.nl)

Carolus J. Reinecke: [carools.reinecke@nwu.ac.za](mailto:carools.reinecke@nwu.ac.za)

**Table of Contents**

| Section 1: Original ^1^H-NMR spectral data for intervention 1 | 3 |
| --- | --- |
| Section 2: Threshold value and normalization | 4 |
| Section 3: Data pre-processing and statistical analysis  Section 3.1: Case reduction  Section 3.2: Data transformation and scaling  Section 3.3: Cross-sectional analysis  Section 3.3.1: Univariate analysis  Section 3.3.2: Multivariate analysis  Section 3.4: RM ANOVA  Section 3.5: Unfolded PCA  Section 3.6: ASCA | 4  5  7  7  7  9  14  15  16 |
| Section 3.7: Graphs on excretion kinetics  Section 3.8: NMR spectra on the excretion of six substances  Section 4: NMR analysis on guanidinoacetic acid | 18  19  20 |
| References | 22 |

**Section 1 Original ^1^H-NMR spectral data for intervention 1**

Intervention 1 refers to consumption of flavored water only – the cause of the benzoic acid biotransformation described in the main paper. The data include urine samples from the 23 experimental cases that completed the study, yielding a total of 138 study samples. The original design was based on 24 cases (individual subjects), although one did not participate in the flavored water experiment. The original ^1^H-NMR spectrum of each of the urine samples analyzed was divided into 0.02-ppm equal-sized bins between 0.5 and 10 ppm, excluding the region of the water peak (4.72–4.88 ppm). This resulted in 467 bins containing spectral data. The original, raw spectral data matrix of all the samples from the vehicle intervention analyzed for this study is given as an electronic file in Excel format (S2 File: CI_unscaled raw vehicle data.xlsx). Table A represents a small extract from this file.

|  |  |  |  | **Bin** | **1** | **2** | **3** | … | **466** | **467** |
| --- | --- | --- | --- | --- | --- | --- | --- | --- | --- | --- |
|  | **Sample** |  |  | **No.** | **9.99** | **9.97** | **9.95** | … | **0.53** | **0.51** |
| **Batch** | **name** | **Case** | **Treatment** | **Time** |  |  |  |  |  |  |
| 1 | F41-1 | F | Vehicle | –1 | 252890.2 | 171823.4 | 163215.5 |  | 384216.4 | 266010.8 |
| 1 | F410 | F | Vehicle | 0 | 151299.1 | 100954.3 | 86908.15 |  | 262467 | 216768.2 |
| 1 | F411 | F | Vehicle | 1 | 79268.87 | 105579.2 | 95048.77 |  | 76325.53 | 126937.3 |
| 1 | F412 | F | Vehicle | 2 | 84927.78 | 101751.3 | 127028.2 |  | 77872.51 | 68765.72 |
| 1 | F413 | F | Vehicle | 3 | 74747.86 | 144056.7 | 47937.61 |  | 91158.41 | 58079.11 |
| 1 | F414 | F | Vehicle | 4 | 67488.71 | 70024.98 | 68237.62 |  | 103052 | 89226.37 |
| 2 | E51-1 | E | Vehicle | –1 | 120268.5 | 125437.8 | 123530.6 |  | 143314 | 122366.1 |
| 2 | E510 | E | Vehicle | 0 | 206635.9 | 142868.1 | 112930.5 |  | 198141.4 | 156402.8 |
| 2 | E511 | E | Vehicle | 1 | 106837.9 | 97280.69 | 132238.9 |  | 168890.4 | 128759.2 |
| 2 | E512 | E | Vehicle | 2 | 88524.67 | 110966.2 | 92268.4 |  | 75853.71 | 102751.3 |
| 2 | E513 | E | Vehicle | 3 | 101023.8 | 97107.34 | 59599.65 |  | 76011.03 | 88203.6 |
| 2 | E514 | E | Vehicle | 4 | 134874.8 | 93287.56 | 67920.9 |  | 182858.7 | 85690.59 |
| 3 | G41-1 | G | Vehicle | –1 | 183044.6 | 110147.5 | 92701.17 |  | 368924.5 | 308433.9 |
| 3 | G410 | G | Vehicle | 0 | 220505.4 | 134572.8 | 99752.4 |  | 451830.2 | 324699.9 |
| 3 | G411 | G | Vehicle | 1 | 229915.6 | 116229.6 | 130475.5 |  | 951134.8 | 407340.4 |
| **Table A: Raw ^1^H-NMR spectral data given as an electronic file in Excel format (see S2 File: CI_unscaled raw vehicle data.xlsx) attached online as part of the Supporting Information** | | | | | | | | | | |

**Section 2 Threshold value and normalization**

The use of NMR spectroscopy as a tool for metabolomics is limited by the sensitivity (limit of detection) of NMR [1], requiring an approach in discarding noise while retaining and then measuring real peaks. The spectral intensities within the original 467 bins generated here ranged from 7.8 x 10^5^ to 1.7 x 10^9^. Based on previous empirical experience with NMR spectral analysis, we defined a threshold value of 2 x 10^6^, being approximately the limit of detection of metabolomic substances presumed to be present in a spectral bin. To reduce the uncertainty in the data at the level of the approximate detection limit, we applied the threshold of 2 x 10^6^ to the data matrix, setting all values below this threshold to zero. We subsequently applied a “zero-filter” to remove bins with more than 50% zero observations across all six time points, as applied for fusion of MS-based metabolomics data [2]. This resulted in a reduction in the number of bins containing spectral data from 467 to 347. To account for the dilution differences between the urine samples, the value of each bin was made relative to the CH_2_ peak of creatinine. This peak is contained in the 4.05 and 4.07 ppm bins. Therefore, each bin value was divided by the sum of the value in these two bins to normalize the bin values relative to creatinine. After normalization, the three bins related to creatinine (4.05, 4.07 and 3.05 ppm) were also removed from the matrix, giving a final total of 344 bins containing spectral data. The above processing was performed in Matlab [3] as outlined in the script provided as additional material.

**Section 3 Data pre-processing and statistical analysis**

The data resulting from this intervention study potentially contain volumes of information. However, unlocking it is a challenge for various reasons, the first being the presence of three major sources of variation: (i) the four experimental treatments; (ii) the variation over time; and (iii) the variation between cases participating in the study (potentially the greatest source of variation). The second relates to the analytical platform used. NMR spectra using equidistant binning results in hundreds of bins, in this instance 467. This far exceeds the number of cases measured (23), which is known to cause more traditional statistical methods such as MANOVA (multivariate analysis of variance) to fail [4]. Third, the quantified values may be very low for some bins and carry large uncertainty as to the accuracy of the value reported. As a result, values below a certain threshold are converted to zeros (Section 2 above), which again poses a challenge for many statistical methods making an assumption about distribution.

The design of the study aimed to alleviate these challenges to some extent by (i) repeatedly measuring the same individuals across both time and treatment factors; (ii) selecting cases with similar demographics, i.e. age and living conditions; and (iii) limiting the influence of dietary difference by exposing cases to each intervention while in a fasting state. Finally, the results discussed in this paper pertain only to a subset of the data, one of the four treatment interventions, making this initial analysis more palatable yet still highly informative.

Taking into account the design of the experiment as well as the limitations of the various statistical techniques, a range of methods were applied to this subset of the data, as discussed in more detail below.

**Section 3.1 Case reduction**

Reproducibility and repeatability are not known concerns in NMR analysis [5]. Nevertheless, three pooled quality control (QC) samples were included in each NMR batch run, in the first, middle and last positions. The role of the QC samples was not to correct for any drift or between-batch effects, as done in GC-MS for instance, as this category of confounding factors should not be present in NMR analysis. Rather, the QC samples were used to identify batches which produced unexpected and therefore untrustworthy results due to some external factor we did not or could not control or measure. Outlier detection was based on the scores from a principal component analysis (PCA) with 90% confidence regions, along with Hotelling’s T^2^ statistic (Figure A). A two-component PCA model was constructed based on the log-scaled and centered normalized data (464 bins) from only the QC samples. Note that the 50% zero filter was not yet applied as some of the zero measurements may be due to outlying batches.

All three QC samples from batches 2 and 3 were identified as outliers. This could have been due to some analytical error which occurred during the preparation of the samples from these two batches. This chemical shift in the NMR spectra of the samples in these two batches, however, made it difficult to compare the peaks in these samples with those of the samples from the 21 other batches. Consequently, these two batches were excluded from further analysis as batch corrections based on only three QCs may introduce more error than they remove. We could eliminate these batches with little effect as all samples from a given case represented a batch; so, even though we sacrificed in terms of sample size, the exclusions did not change the design.

| **A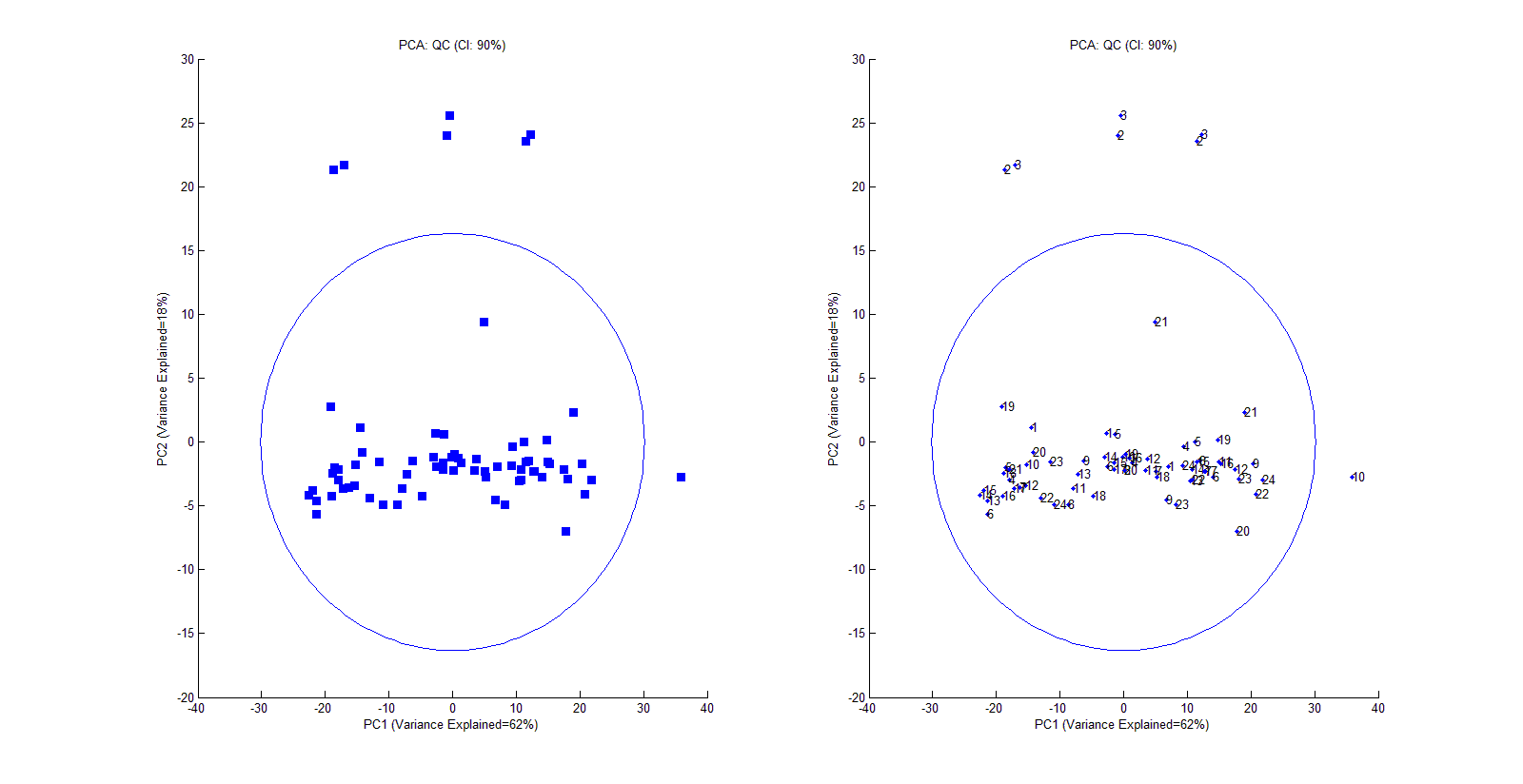** | **B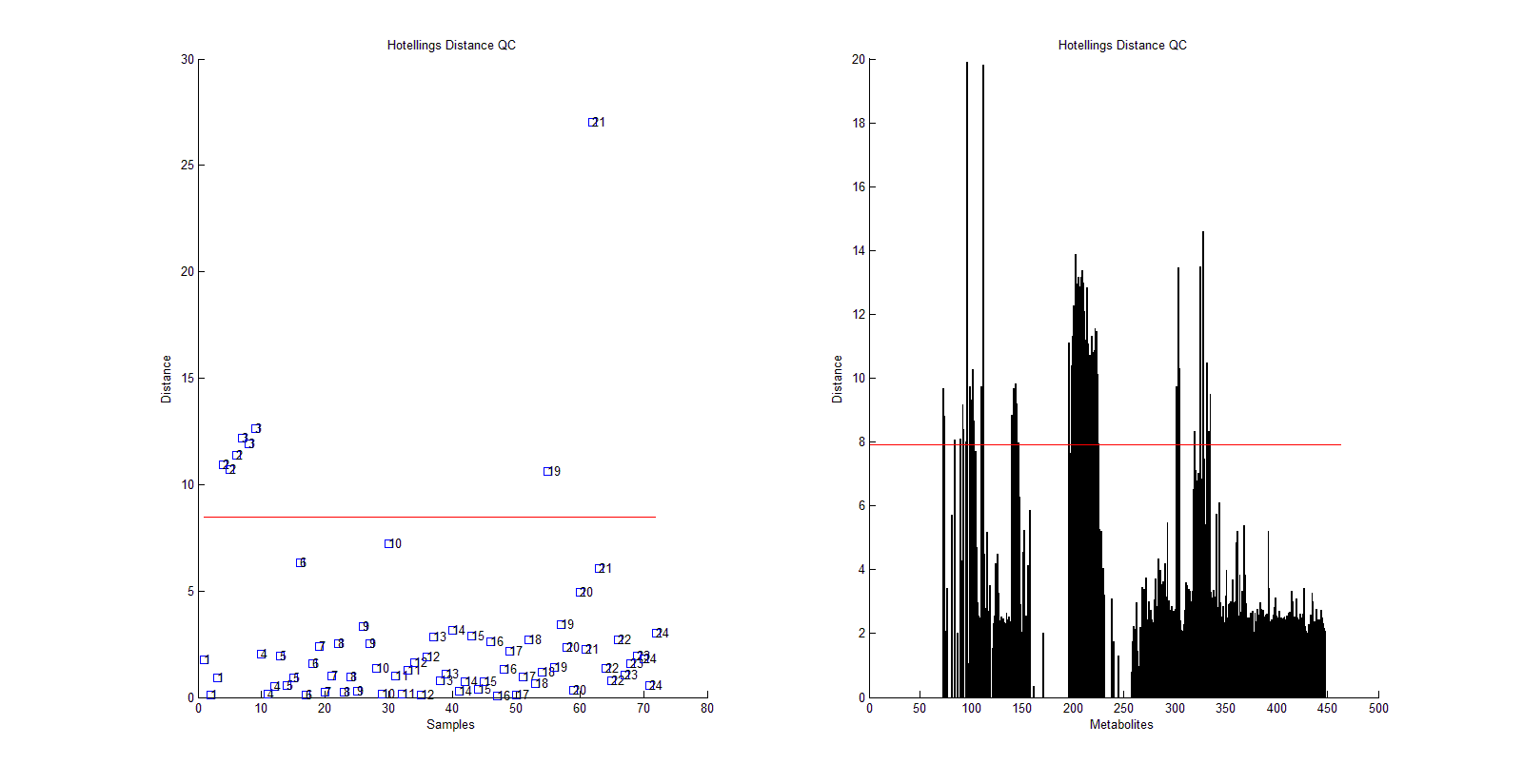** |
| --- | --- |
| **Figure A: QC Outlier Detection**  The figure shows the results of a PCA analysis on the QC samples. The PCA scores of all three QC samples for batches 2 and 3 fall outside the 90% confidence region (A). The Hotelling’s T^2^ statistics for these same samples flag them as outliers (B). Two other QC samples from different batches were also flagged; however, as only one of the three QCs in these batches was flagged, these batches were not removed. | |

The above processing and analysis was performed in Matlab using PCA function provided by the PLS Toolbox [3], as outlined in the script provided as additional material.

**Section 3.2 Data transformation and scaling**

To reduce the uncertainty in the data, we applied a threshold to the data matrix followed by a “zero-filter” (Section 2). The reduced data were log transformed to ensure that the additive models used to extract information from the data were appropriate [6]. Log transformation also makes skew distributions – often resulting when variables (i.e. bins) assume only positive values, such as here – more symmetric. Symmetry is assumed in many statistical methods, including some of the methods used here. The above processing was performed using Matlab.

**Section 3.3 Cross-sectional analysis**

Here we made use of various methods to compare times –1, 1, 2, 3 and 4 hours with time 0. We did this to answer the following questions:

1. At what time did the effect of the vehicle first become apparent?
2. At what time did the effect of the vehicle start to diminish?
3. Were individuals in a fasting state more similar?
4. Did their metabolism change greatly while fasting?

While the last two questions do not relate to the aims of the study, their answers influence how we interpret other results. Next, we discuss the univariate methods used. These analyses were performed in Matlab and included (i) univariate statistics using the statistics toolbox; (ii) PCA using the PLS Toolbox; and (iii) PLS-DA using the PLS Toolbox [3].

**Section 3.3.1 Univariate analysis**

The Wilcoxon signed rank test was first employed to find significant differences in the average ranks of bin values between two time points. This hypothesis test is non-parametric (i.e. makes no distributional assumptions) and assumes samples are paired. Since cases were measured repeatedly, it is important to use statistical methods that can model this dependence. Paired measures reduce some of the variation that is not of interest (i.e. between cases) and methods exploiting this are therefore better able to detect group differences. All hypothesis tests rely on random sampling, which is not the case here. Convenience sampling, as used here, can bias p-values produced by tests and they should be evaluated with caution. Further multiple testing requires controlling the family-wise error rate. However, since we did not base variable selection on hypothesis testing, these factors were less of a concern. Our aim here was simply to gain insight into the general progression of the effect of the treatment over time (i.e. whether differences became more pronounced or not).

Given the limitations of hypothesis testing listed above, other univariate measures supplemented our evaluation of the general progression. For each bin, two more intuitive measures of group differences were also evaluated: (i) effects sizes based on the Wilcoxon signed rank z-statistic to evaluate practical significance; and (ii) fold change value.

Figure B provides the Volcano plots for all pairwise time point comparisons. A Volcano plot is essentially a scatter plot of the log_2_ scaled fold change values against the –log_10_ scaled p-values (in this case p-values from the Wilcoxon signed rank test). By performing scaling, the plot is able to highlight the discriminatory bins in the upper left and right corners for a given threshold. In this instance bins with p-values below 0.05 and fold change values larger than 2 were highlighted in the figures. From Figure B it is evident that individual bins start to reflect the effect of the vehicle intervention 1 hour after consumption.

| 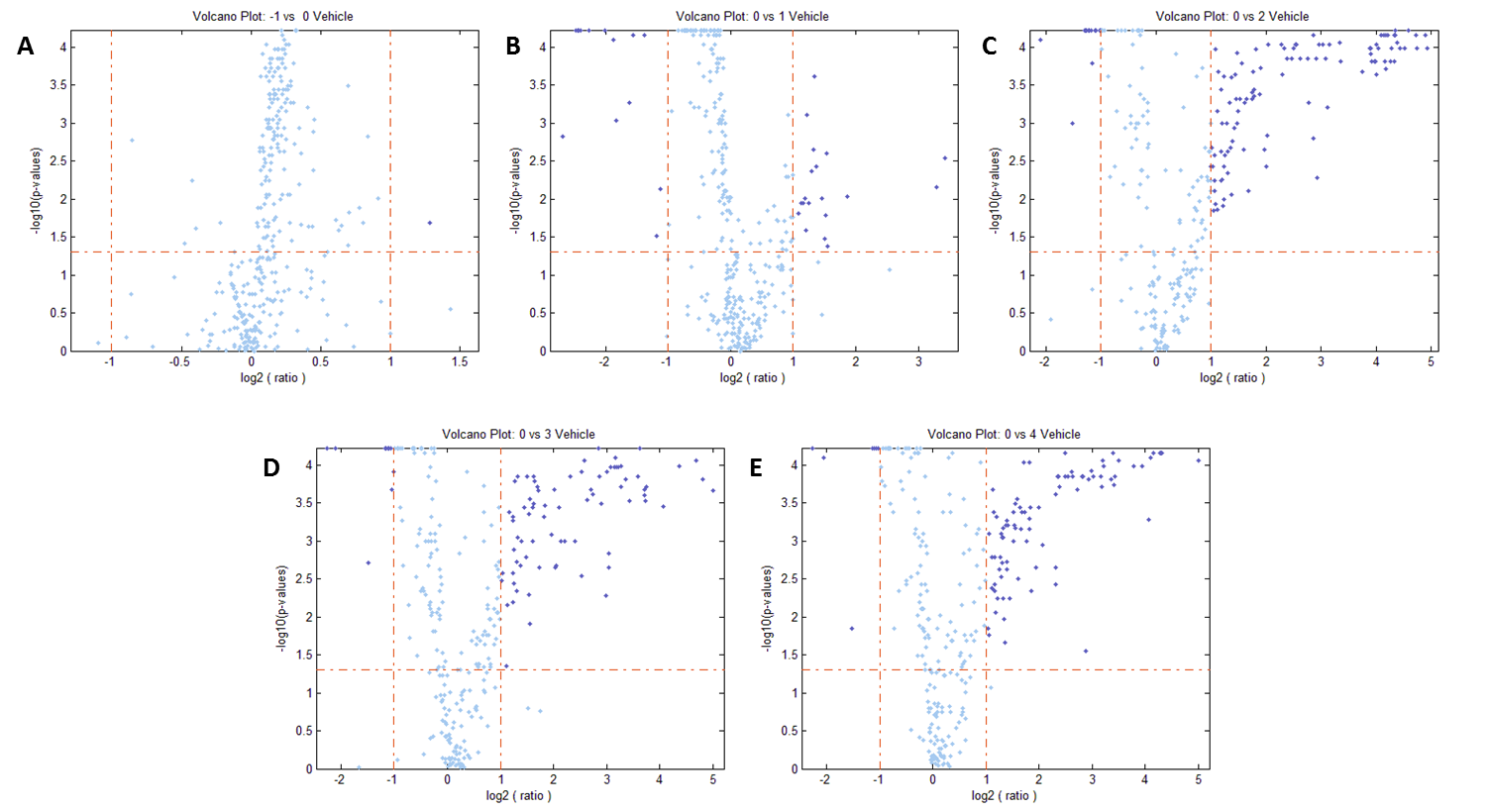 |
| --- |
| **Figure B: Volcano Plots of Pairwise Time Point Comparisons with time 0**  The figure displays the individual Volcano plots for the pairwise comparisons of time –1 vs. time 0 (A); time 0 vs. time 1 (B); time 0 vs. time 2 (C); time 0 vs. time 3 (D); and time 0 vs. time 4 (E). |

**Section 3.3.2 Multivariate analysis**

To gain insight into the interactions between bins, i.e. to find bins which in combination differ between groups, we made use of multivariate statistical methods. Euclidean and Ward cluster analysis was used to find natural groupings between the cases based on the bin data provided, but without providing information on the known group structure (unsupervised). Cluster analysis makes use of a distance measure (Euclidean) to measure the distances or difference between samples based on the measured variables. A linkage method (Ward) was then used to group neighboring samples. The groups formed can be viewed using a dendrogram. If these groups coincide with the known group structure (as indicated by labeling the dendrogram after the fact), we can be confident that the data set contains discriminatory bins. For more detail on cluster analysis refer to Field [7]. Figure C shows the dendrograms for all pairwise time point comparisons with time 0. The interesting findings from these dendrograms are discussed in the main paper.

| 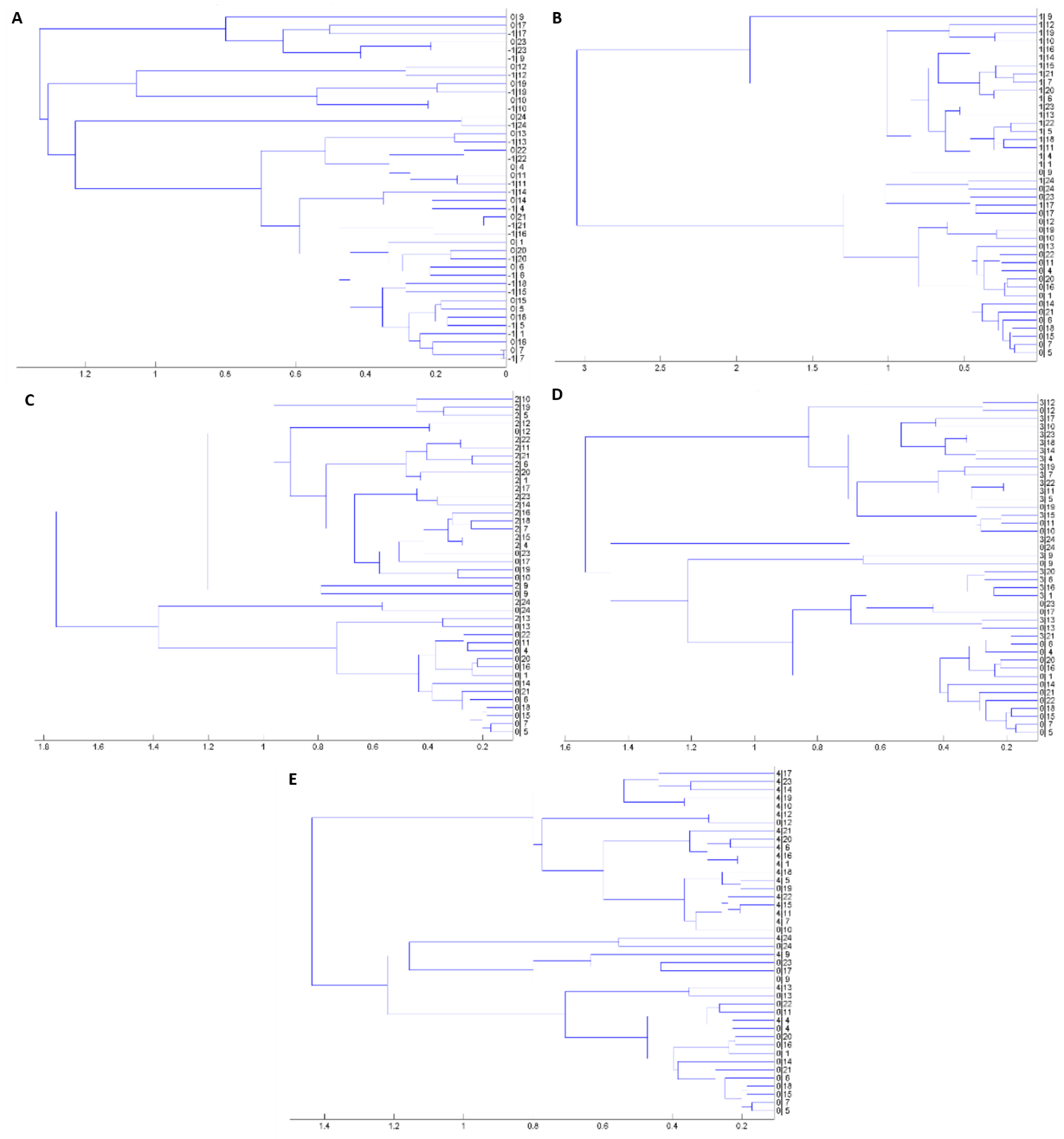 |
| --- |
| **Figure C: Dendrograms of Pairwise Time Point Comparisons with time 0**  The figure displays the individual dendrograms for the pairwise comparisons of time –1 vs. time 0 (A); time 0 vs. time 1 (B); time 0 vs. time 2 (C); time 0 vs. time 3 (D); and time 0 vs. time 4 (E). |

Principal component analysis (PCA) was used to project the hundreds of bins onto a two-dimensional subspace using the following model:

If $D$ represents a data matrix of $N$ cases (i.e. samples) by $K$ variables, where the $N$ cases represent observations across all factor levels, then the PCA model of $D$ can be written as: $D=TP'+E$, where $T$ represents the scores and $P’$ the transposed loadings matrices, respectively. Given that the dimensionality of $D$ is reduced, the error matrix $E$ is added to account for the variation not explained by the model ($TP’$). For more detail refer to [8].

Such a projection allows for a clearer view of the influence of experimental factors, specifically which factor dominates the variability in the data. PCA, like cluster analysis, is also an unsupervised method, therefore the influence of experimental factors becomes visible when labeling the scores (values for samples in the new subspace) according to associated factor level after the model has been constructed, in our case the times associated with each sample. Projecting the matrix also allows us to evaluate the influence of each variable on the projected scores. In other words, if we find that our group structure predominantly explains the covariance structure of the data, we can evaluate the loadings (values for variables or bins in the new subspace) to identify a list of variables with discriminatory ability on their own or in combination. The loadings were not used during the PCA analysis but extensively so in the ASCA model (discussed later), which is built on the PCA model.

Figure D shows the score plots for all pairwise time point comparisons with time 0. Figure D(A) indicates how fasting state did not change over time since times –1 and 0 are very similar and the individual cases are closely comparable. Figures D(B) – D(E) show how the global bin profile changes relative to the starting point of the intervention. The effect of the vehicle is already evident after the first hour, starts to diminish after two hours, and cases continue to return to normal three and four hours after the intervention.

| 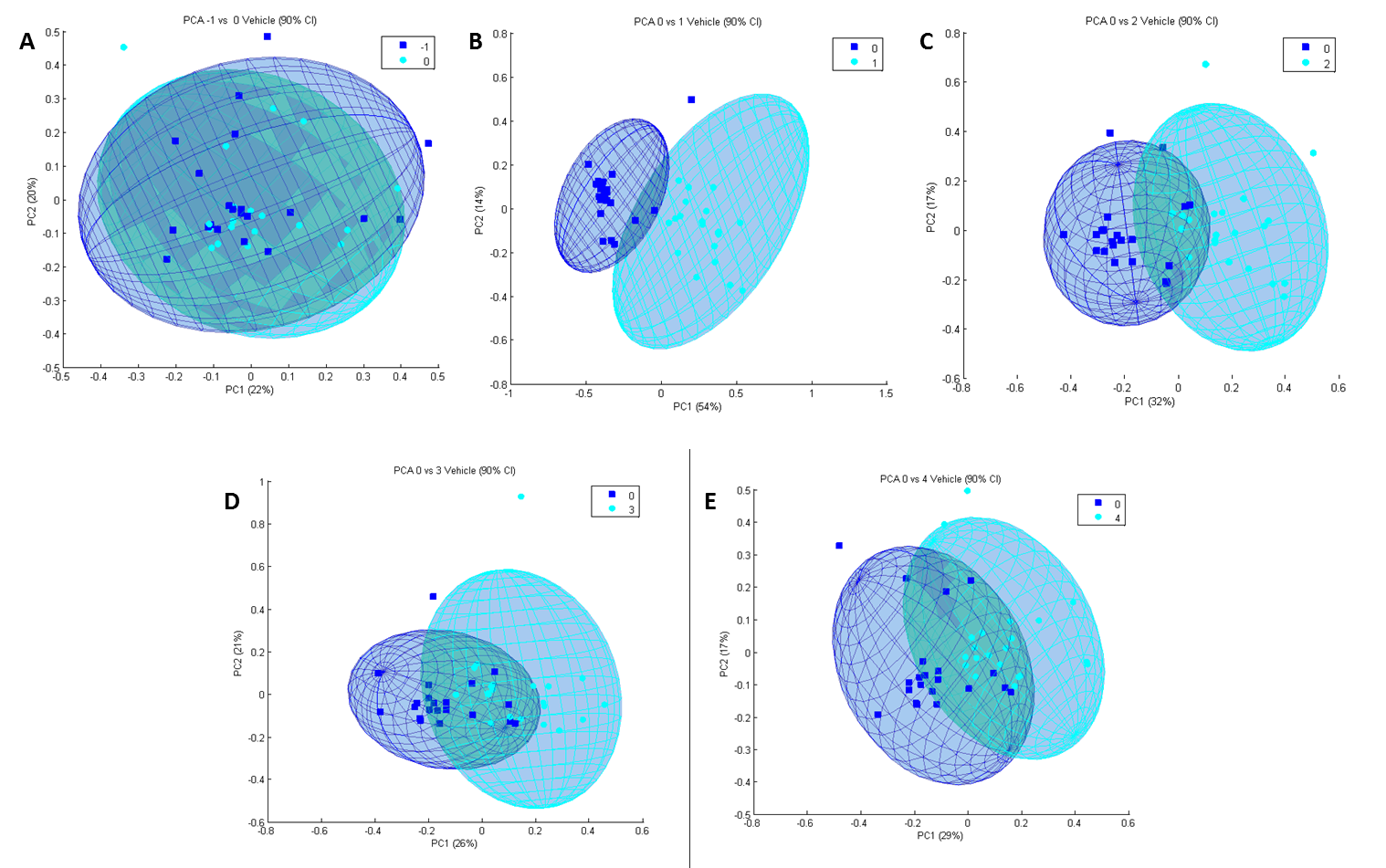 |
| --- |
| **Figure D: PCA Score Plots of Pairwise Time Point Comparisons with time 0**  The figure displays the individual score plots of the PCA models for the pairwise comparisons of time –1 vs. time 0 (A); time 0 vs. time 1 (B); time 0 vs. time 2 (C); time 0 vs. time 3 (D); and time 0 vs. time 4 (E). |

Lastly, partial least-squares discriminant analysis (PLS-DA) was performed. This method is similar to PCA but makes use of the known group structure (i.e. it is a supervised method). The benefit of using this information is that the method is better able to identify a list of discriminatory variables. The downside is that the model itself may not generalize to other data sets as it is known to overfit [9]. Since our aim was not to develop a classification model, we were not too concerned about this. However, we do report two measures of confidence in the bins selected: (i) R^2^ to measure the goodness of the model fit; and (ii) Q^2^, the leave-one-out cross-validated R^2^, to assess generalizability or sensitivity to the individual samples used to construct the model. Figure E shows the score plots for all pairwise time point comparisons with time 0, whereas Table B contains the fit statistics. We can draw the same conclusion as from the PCA, but with a more pronounced difference, which is to be expected given that PLS-DA is a supervised method.

| 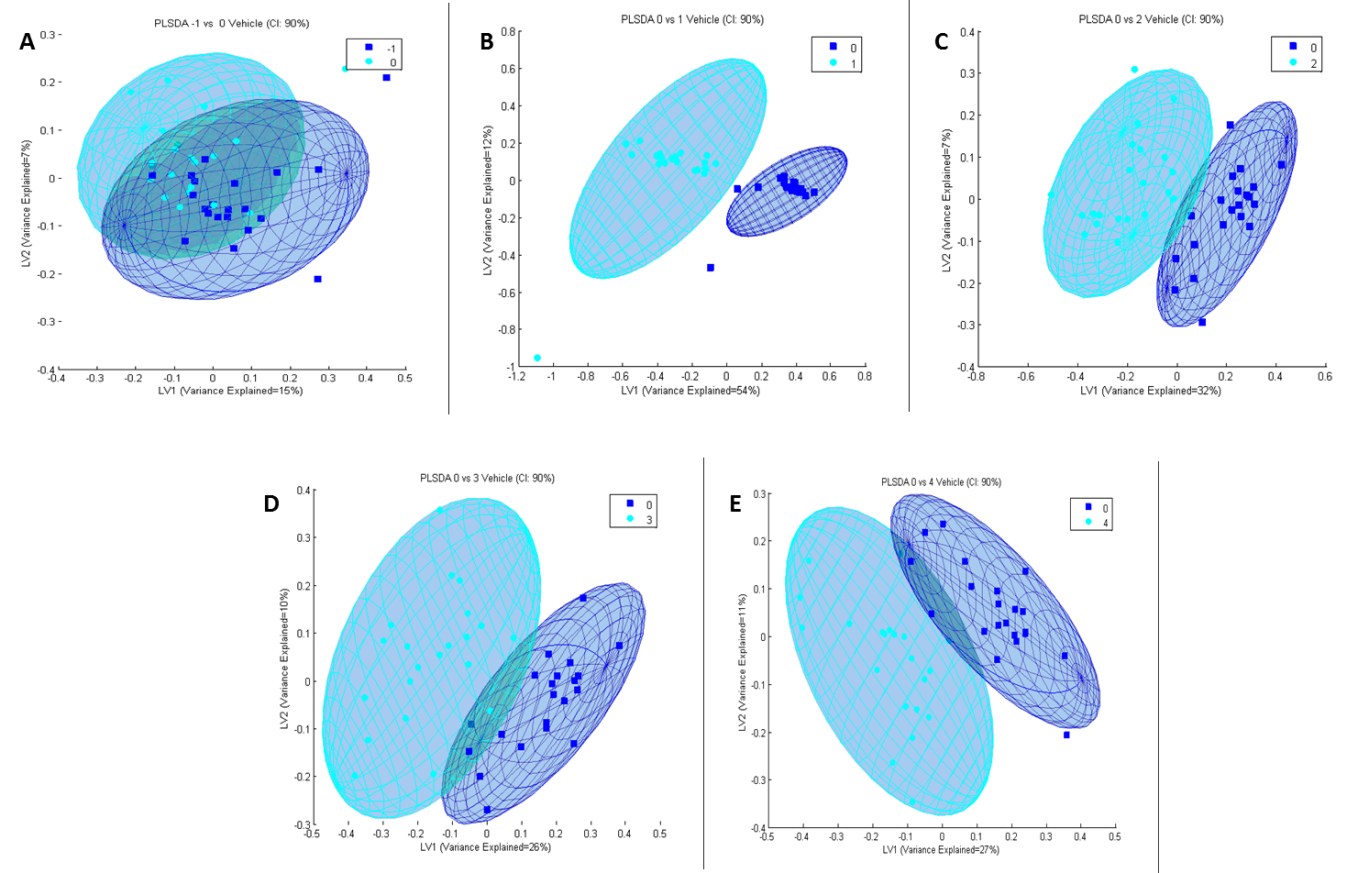 |
| --- |
| **Figure E: PLS-DA Score Plots of Pairwise Time Point Comparisons with time 0**  The figure displays the individual score plots of the PLS-DA models for the pairwise comparisons of time –1 vs. time 0 (A); time 0 vs. time 1 (B); time 0 vs. time 2 (C); time 0 vs. time 3 (D); and time 0 vs. time 4 (E). |

| **Comparison** | **R^2^** | **Q^2^** |
| --- | --- | --- |
| **Time 0 vs. –1** | 0.171 | 0.002 |
| **Time 0 vs. 1** | 0.776 | 0.836 |
| **Time 0 vs. 2** | 0.726 | 0.704 |
| **Time 0 vs. 3** | 0.646 | 0.488 |
| **Time 0 vs. 4** | 0.628 | 0.501 |
| **Table B: PLS-DA Fit Statistics Pairwise Time Point Comparisons with time 0**  The table reports the fit statistics for the first latent variable of PLS-DA models for the pairwise comparisons. | | |

The results from the cross-sectional analysis led us to conclude that, while between-case variation is significant, we were still able to find bins that differ from their baseline measure (time 0). We also found that the fasting state was long enough to reduce between-case variation (time –1 vs. 0). We made use of the bins selected by the PLS-DA models as one of the shortlists to be included in the Venn analysis that produced the key metabolites quantified and discussed. We wanted to include bins in this list that were informative at any point in time (compared to the baseline, i.e. time 0) while accounting for the multiple PLS-DA models constructed. As such we decided to include bins with VIP values exceeding 2 in any of the models. A VIP score is calculated for each variable in the data matrix and provides an indication of the variable’s importance in the projection (hence abbreviated as VIP). For more detail on this calculation refer to [10].

**Section 3.4 RM ANOVA**

RM ANOVA (repeated measures analysis of variance) is a statistical method that deconstructs a data set into the known sources of variation in order to assess which of these sources significantly influenced the values recorded in the data set. This deconstruction is performed by calculating and subtracting factor level means (ANOVA) – in our case the means for the time points, as well as for each case since the same cases were measured across all time points. The primary aim of this analysis was to identify variables that changed significantly in time (across the six time points). We constructed the ANOVA model as follows:

Let$X$ represent all observations for a given bin and let $\alpha$ and $\beta$ represent the Time and Case factors, respectively. We can model the change in time as follows:

$$X_{ij}=\mu+\alpha_{i}+\beta_{j}+{\alpha\beta}_{ij}$$

where $\mu$ represents the overall mean of the bin

$\alpha_{i}$ represents the time effects with $i=1\ldots6$

$\beta_{j}$represents the case effects with $j=1\ldots21$

${\alpha\beta}_{ij}$ represent the random interaction effect between case *i,* treatment *j*, i.e. the random error not explained by the model.

Therefore $X_{ij}$ represents the observation on the bin for case $j$, $i-2$ hours after consuming the vehicle, as modeled on the basis of the factor means and random noise. Bins for which the factor means play a larger role than the random noise to obtain the observed value are flagged as significant.

RM ANOVA essentially expands the cross-sectional approach to account for the complete study design of the data set (or subset since we retain only time as a factor). However, it does not account for interactions between bins as it is a univariate method. RM ANOVA can be expanded to evaluate multiple variables, but breaks down when the number of variables exceeds the number of cases, as mentioned earlier. For in-depth insight into ANOVA methods, refer to Tabachnick & Fidell [11,12]. Here we used RM ANOVA to identify individual bins that changed significantly in time, as constituting the second shortlist to be included in the Venn analysis. To control the family-wise error rate resulting from applying RM ANOVA hypothesis testing multiple times, bins were selected only if their associated p-values were estimated as 0. This analysis was performed in Matlab using the statistics toolbox [3] and code adapted from Trujillo-Ortiz et al [13].

**Section 3.5 Unfolded PCA**

Unfolded PCA was applied to gain insight into the global (i.e. multivariate) effect of the vehicle over time. This method extends the RM ANOVA idea to the multivariate setting. However, it does not explicitly take the study design into account as it does not truly decompose the data into the known sources of variation. The data tensor (with three dimensions representing cases, times and bins) was unfolded in time as illustrated in Figure F Unfolding transforms a three-dimensional tensor into a two-dimensional matrix, allowing for principal component analysis. PCA of the unfolded tensor provides insight into the effect of the vehicle in time on the bins (indicated by the ellipses and centroids) as well as individual cases (indicated by the overlaid trajectories) (Figure 5 in the main paper). For a more in-depth discussion on unfolding refer to [14]. This analysis was performed in Matlab using the PCA function of the PLS toolbox.

| **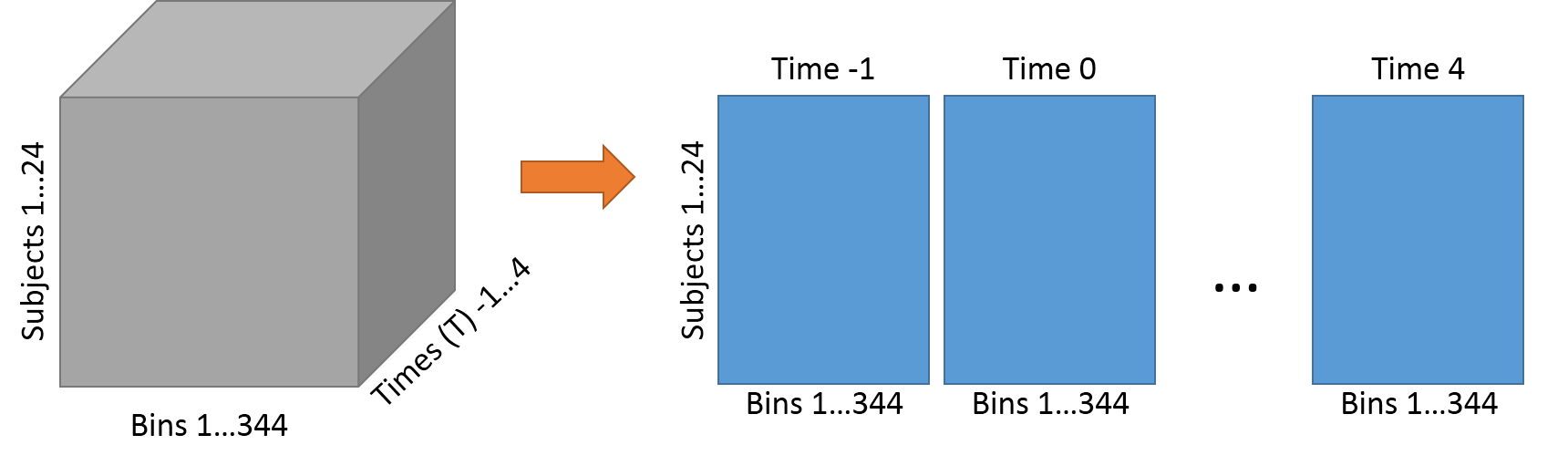** |
| --- |
| **Figure F: Unfolding Data**  The figure illustrates how a new data matrix was formed by unfolding the data tensor, generated by the vehicle intervention, over time. |

**Section 3.6 ASCA**

ASCA (ANOVA-simultaneous component analysis) was developed for analyzing designed metabolomics data. It can not only account for the study design but also for the high-dimensionality of the data. In essence ASCA combines MANOVA and PCA (as can be deduced from the name) by first deconstructing the data as done in ANOVA and subsequently applying PCA to each subset. The model can be summarized as follows:

If $D$ represents a data tensor then ASCA will deconstruct $D$similarly to RM ANOVA:

$$\boldsymbol{D}=\boldsymbol{\mu}+\boldsymbol{\alpha}_{\boldsymbol{i}}+\boldsymbol{\beta}_{\boldsymbol{j}}+\boldsymbol{\alpha\beta}_{\boldsymbol{ij}}$$

Note the adoption of boldface symbols, as the factor now represents matrices of means across all variables as opposed to just a vector of means for one variable for each factor level. Next, PCA is performed for each matrix

$$D=1m+T_{\alpha}P_{\alpha}^{'}+T_{\beta}P_{\beta}^{'}+T_{\alpha\beta}P_{\alpha\beta}^{'}$$

For an in-depth discussion on the ASCA method, refer to Jansen *et al.* [15] and Smilde *et al.* [16]. As mentioned in the PCA discussion, we can evaluate the loadings (values for variables in the new subspace) to identify a list of bins with discriminatory ability on their own or in combination. The sum of the squared loadings (SSL) for the first two latent variables was used to identify perturbed bins because in combination they explained 88% of the variation. Bins were seen to have discriminatory ability if their SSL values were above 0.01, the threshold identified based on a Scree plot of all SSL scores (Figure G).

| 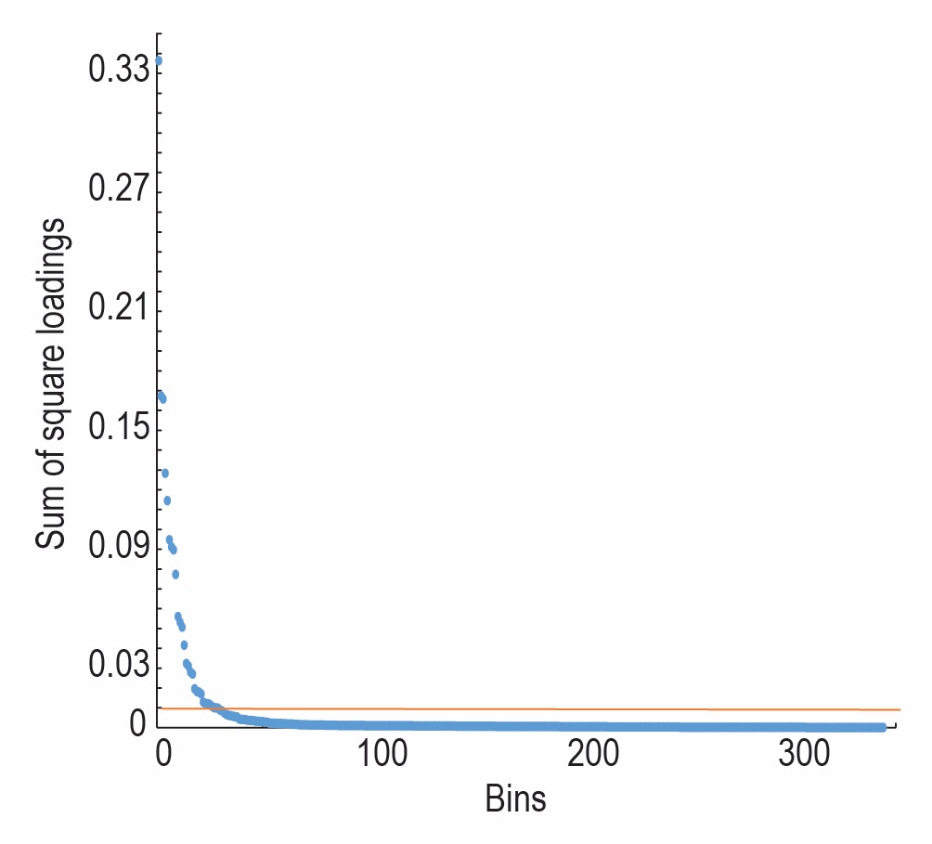 |
| --- |
| **Figure G: Sum of Squared Loadings of ASCA model**  A scree plot of the sum of the squared loadings (SSL) of the ASCA model based on the first two components |

Though this method may have sufficed to identify key bins, as is evident from the Venn results reported in the main paper, it was used in conjunction with the cross-sectional (specifically PLS-DA) and RM ANOVA results to ensure we did not exclude potentially important bins. This analysis was performed in Matlab using the statistics toolbox [3] and code provided by Gooitzen Zwanenburg (available under APACHE Licence 2.0 http://www.apache.org/licenses/LICENSE-2.0.html).

**Section 3.7 Graphs on excretion kinetics**

The graphs shown in Figure H provide a visualization of summary statistics for the quantified data of the metabolically important metabolites summarized in Table 1 of the main manuscript. These plots were generated using notBoxPlot.m a Matlab function developed by Rob Campbell (<http://www.mathworks.com/matlabcentral/fileexchange/26508-raacampbell13-notboxplot>). The raw data is scattered over a 95% confidence interval (red) and 1 standard deviation of the mean (blue).In two cases, outliers of more than 1.5 times the interquartile range were excluded from the figures and the calculations (Hippuric acid 4250 and creatine of 2500 μmoles metabolite/mmole creatinine, both observed at 1 hour following the intervention).

Reaction kinetics are represented by concentration–time profiles of the six metabolites for the 22 participants that participated in the intervention, shown at times 1, 2, 3 and 4 hours following the intervention (time 0 hour). All quantified values of metabolites in all samples were normalized relative to the creatinine concentration recorded for each sample.

**A:** **B:**


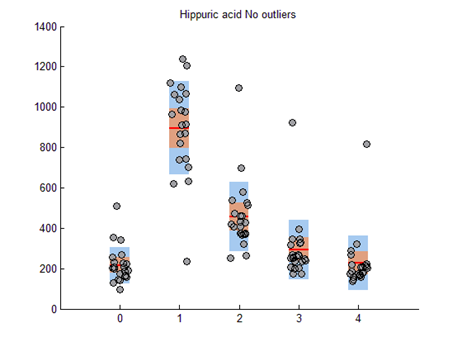

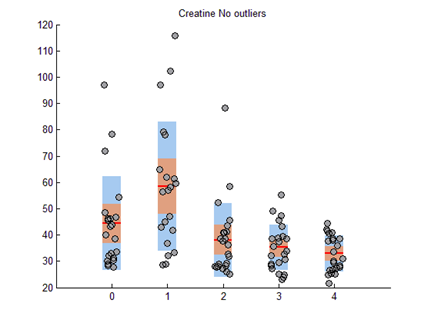


**C:** **D:**


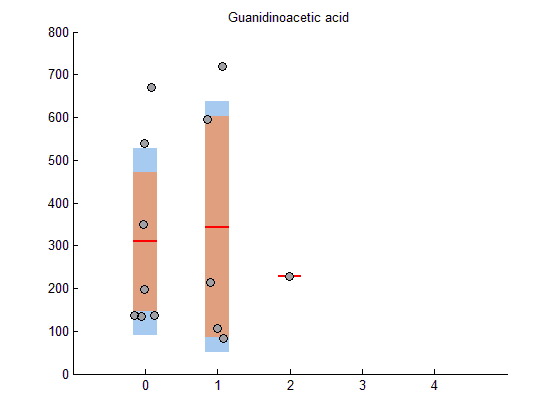

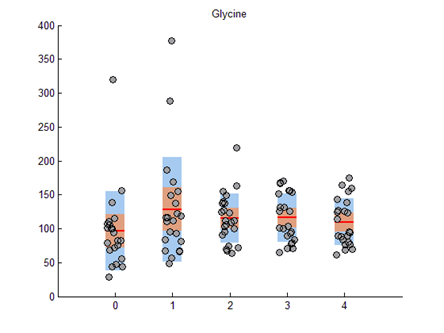


**E:** **F:**


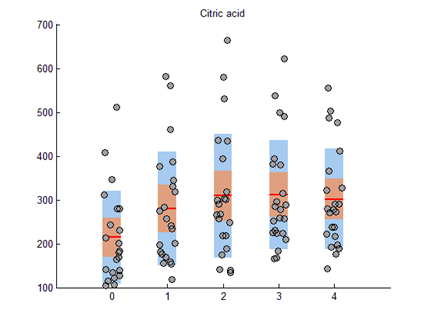

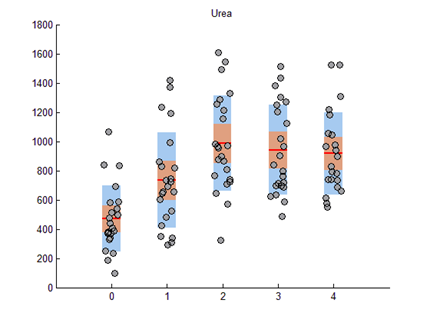


**Figure H: Urinary Excretion kinetics of important metabolites**

Concentration–time profiles of hippuric acid (A), creatine (B), guanidinoacetic acid (C), glycine (D), citric acid (E) and urea (F) for times 1, 2, 3 and 4 hours following consumption of 500 ml benzoic acid containing flavored water at time 0 hour. All samples contained all metabolites, except for guanidinoacetic acid, as indicated in (C).

**Section 3.8 NMR spectra on the excretion of six substances**

The authentic spectra and power values from time dependent PCA’s were used to re-analyze the outcomes of the intervention. It appeared that three unknown substances (Figure I: (A) 8.22 ppm, (B) 6.485 ppm and (D) 3.075 ppm), as well as methylguanidine (2.83 ppm - shown in Figure I(C)) — a known endogenous metabolite, changed due to the intervention. Methylguanidine is synthesized from creatinine concomitant with the synthesis of hydrogen peroxide from endogenous substrates in peroxisomes. It will be speculative to indicate its link to the biotransformation study, but it may be linked to oxidative stress. Although none of the four substances were identified as VIP’s in the PLS-DA, their concentrations changed in time following the intervention. As such, they could contribute to the centroid values returning towards the value observed before the intervention, without reaching the exact value of time 0. These observations indicate that the consequence of benzoic acid consumption is more complex than just its biotransformation to hippuric acid.


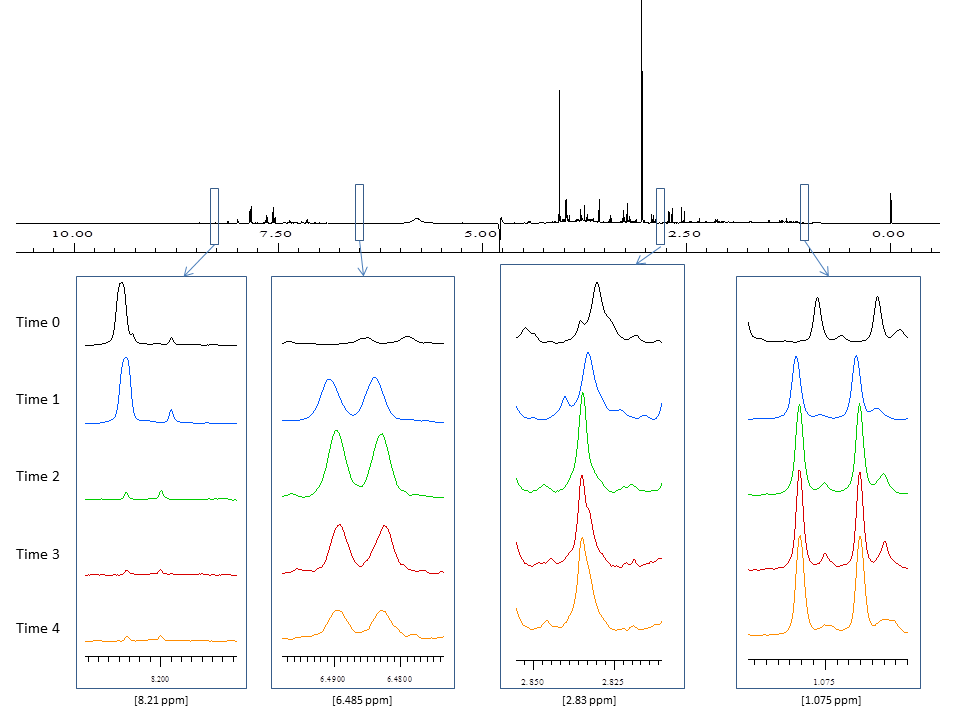


**Figure I: 500 MHz 1H-NMR spectra of minor components from urine.** Top spectrum taken from a urine sample of one selected case; spectra from left to right are: (A) unknown (8.11 ppm), (B) unknown (6.485 ppm), (C) methylguanidine (2.83 ppm) and (D) unknown (1.075 ppm). The substances for times 0 (black), 1 (blue), 2 (green), 3 (red) and 4 (orange).

**Section 4 NMR analysis on guanidinoacetic acid**

Theoretically, the chemical structure of guanidinoacetic acid contains three carbon atoms of which only one has free hydrogen atoms (-CH2-) that would produce a singlet in the NMR spectrum. This is observed in an example for one of our cases, as shown in the one dimensional (1D) spectrum below. Since guanidinoacetic acid does not have any correlating protons, COrrelation SpectroscopY (COSY) two dimensional analyses is not applicable. In the spectra shown in Figure J we compared the experimental spectrum from one of our samples (in green) to that of the pure guanidinoacetic acid standard (in red) – both in 1D and 2D ^1^H J-Resolved (JRES) NMR Spectroscopic analysis. The results indicate that the experimental spectrum lines up exactly to the guanidinoacetic acid singlet (3.77 ppm), confirming the identity of guanidinoacetic acid in the urinary sample.


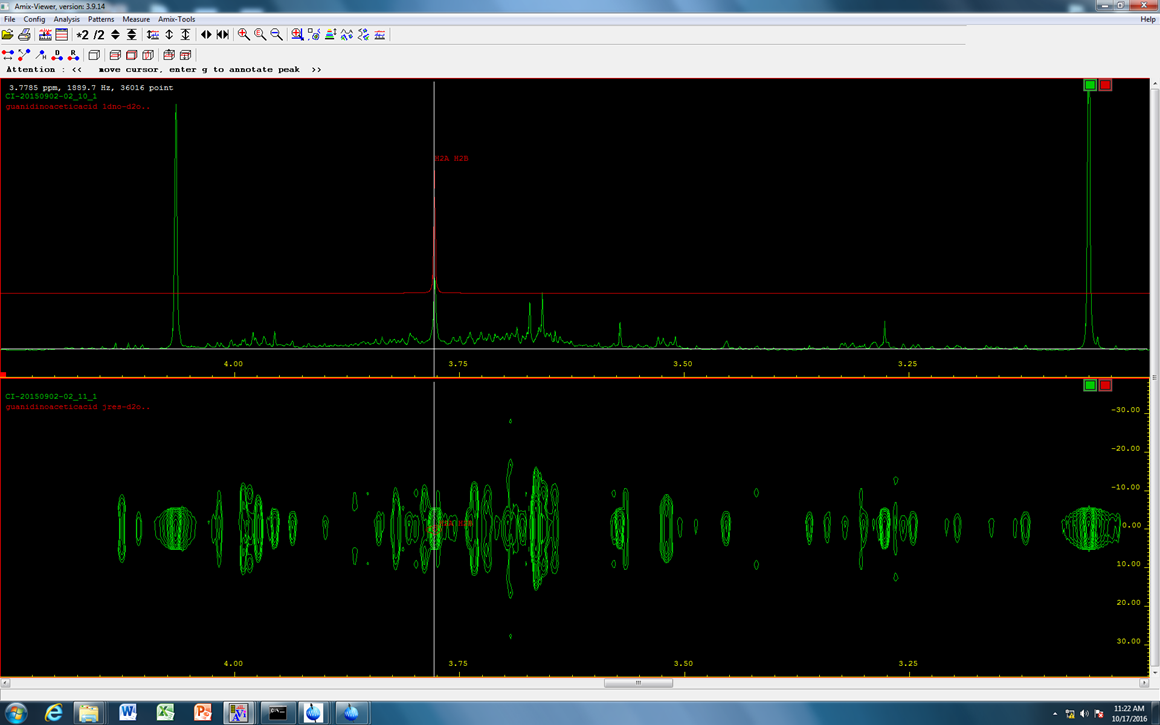


**Figure J: 1D and 2D JRES NMR spectra**

Analysis of the spectra from a urine sample shown in green and from a guanidinoacetic acid standard shown in red.

**References**

1. Krojanski HG, Lambert J, Gerikalan Y, Suter D, Hergenröder R. Microslot NMR probe for metabolomics studies. Anal Chem. 2008; 80:8668-8672.
2. Smilde AKM, Van der Werf M, Bijlsma S, Van der Werff-van der Vat BJC, Jellema RH. Fusion of mass spectrometry-based metabolomics data. Anal Chem. 2005; 77:6729-6736.
3. MATLAB with Statistics Toolbox Release 2012b, The MathWorks Inc., Natick, MA, USA and PLS Toolbox 7.0 (2012), Eigenvector Research.
4. Zwanenburg G, Hoefsloot HCJ, Westerhuis JA, Jansen JJ, Smilde AK. ANOVA–principal component analysis and ANOVA–simultaneous component analysis: a comparison. J Chemometrics. 2011; 25:561-567.
5. Viant M, Bearden DW, Bundy JG, Burton IW, Colette TW, Ekman DR, et al. International NMR-based environmental metabolomics intercomparison exercise. Environ Sci Technol. 2009; 43:219-225.
6. Van den Berg RA, Hoefsloot HCJ, Westerhuis JA, Smilde KA, Van der Werf MJ. Centering, scaling, and transformations: improving the biological information content of metabolomics data. BMC Genomics. 2006; 7:1-15.
7. Field AP. Discovering statistics using IBM SPSS Statistics: and sex and drugs and rock 'n' roll. 3rd ed. London: Sage Publications; 2009.
8. Johnson RA, Wichern DW. Applied Multivariate Statistical Analysis. 5th ed. Englewood Cliffs, New Jersey: Prentice Hall; 2002.
9. Westerhuis JA, Hoefsloot HCJ., Smit S, Vis DJ, Smilde AK, Van Velzen EJJ, et al. Assessment of PLSDA cross validation. Metabolomics. 2008; 4(1):81-89.
10. Pérez-Enciso M, Tenenhaus M. Prediction of clinical outcome with microarray data: a partial least squares discriminant analysis (PLS-DA) approach. Hum Genet. 2003; 112:581-592.
11. Tabachnick BG, Fidell LS. Using Multivariate Statistics. 5th ed. Boston: Allyn & Bacon; 2006.
12. Tabachnick BG, Fidell LS. Experimental Designs using ANOVA. Belmont: Thomson Brooks/Cole; 2007.
13. Trujillo-Ortiz A, Hernandez-Walls R, Trujillo-Perez RA. RMAOV1:One-way repeated measures ANOVA. A MATLAB file. 2004. http://www.mathworks.com/matlabcentral/fileexchange/loadFile.do?objectId=5576.
14. Villez K, Steppe K, De Pauw DJW. Use of unfold PCA for on-line plant stress monitoring and sensor failure detection. Biosystems Engineering. 2009; 103:23-24.
15. Jansen JJ, Hoefsloot HCJ, Van der Greef J, Timmerman ME, Westerhuis JA, Smilde AK. ASCA: analysis of multivariate data obtained from an experimental design. J Chemometrics. 2005; 19:469-481.
16. Smilde AK, Jansen JJ, Hoefsloot HCJ, Lamers R-JAN, Van der Greef J, Timmerman ME. ANOVA-simultaneous component analysis (ASCA): a new tool for analyzing designed metabolomics data. Bioinformatics. 2005; 21(13):3043- 3048.
